# Generation and preclinical characterization of a novel bispecific CD19-TCRgammadelta antibody for the treatment of B cell acute lymphoblastic leukemia

**DOI:** 10.3389/fimmu.2026.1728424

**Published:** 2026-02-27

**Authors:** Joseph Kauer, Fabian Vogt, Sebastian Hörner, Valentin Schmidt, Carsten Müller-Tidow, Simon Raffel, Helmut R. Salih, Gundram Jung, Martin Pflügler

**Affiliations:** 1 Department of Immunology, Interfaculty Institute for Cell Biology, University of Tübingen, Tübingen, Germany; 2 Clinical Collaboration Unit Translational Immunology, Department of Internal Medicine, University Hospital Tübingen, Tübingen, Germany; 3 German Cancer Consortium (DKTK), Partner Site Tübingen, a Partnership Between DKFZ and University Hospital Tübingen, Tübingen, Germany; 4 Internal Medicine V, Hematology, Oncology and Rheumatology, Heidelberg University Hospital, Heidelberg, Germany; 5 Cluster of Excellence iFIT (EXC 2180), Image-Guided and Functionally Instructed Tumor Therapies, Eberhard Karls University, Tübingen, Germany

**Keywords:** acute lymphoblastic leukemia, bispecific antibodies, CD19, gammadelta T cells, immunotherapy

## Abstract

**Introduction:**

B-cell acute lymphoblastic leukemia (B-ALL) is characterized by the clonal expansion of immature lymphoblastic cells. Treating patients with disease relapse is challenging, especially after allogeneic stem cell transplantation (aSCT). Although the CD19xCD3 bispecific antibody (bsAb) blinatumomab has improved outcomes for patients with relapsed B-ALL, T cell exhaustion and immune-associated treatment side effects remain problematic. Vγ9Vδ2 T cells constitute a relatively small subset in healthy individuals but their abundance increases after aSCT, and higher numbers correlate with improved outcomes. Unlike ab T cells, Vγ9Vδ2 T cells are not allo-reactive, do not contribute to graft-versus-host disease and release fewer inflammatory cytokines.

**Methods:**

Using hybridoma technology, we here generated a panel of hybridoma-derived monoclonal antibodies directed against the Vγ9Vδ2 receptor that specifically activate Vγ9Vδ2 T cells. Subsequently, we generated an IgG-based recombinant CD19xγδ bsAb to activate Vγ9Vδ2 T cells.

**Results:**

Our bsAb potently induces Vγ9Vδ2 T cell activation, proliferation, lysis of B-ALL cell lines in vitro in a dose-dependent manner. Additionally, the bsAb mediates lysis of primary leukemic blasts of patients ex vivo and depletion of CD19-positive target cells in an autologous setting. No significant alphabeta T cell activation or proliferation was observed.

**Discussion:**

In summary, the selective activation of Vγ9dδ T cells using our novel CD19xγδ bsAb constitutes a promising immunotherapeutic approach for the treatment of B-ALL. Our results warrant further clinical evaluation especially in patients with minimal residual disease after aSCT or CD3-directed bsAb therapy.

## 1 Introduction

B-cell acute lymphoblastic leukemia (B-ALL) is a malignant disease characterized by the clonal expansion of immature lymphoblastic cells. Standard treatment consists of lengthy chemotherapy-based therapy, followed by allogeneic stem cell transplantation (aSCT) for high-risk patients (1, 2). Patients with relapsed or refractory (R/R) B-ALL have a dismal prognosis (3). Salvage treatment for patients relapsing after aSCT remains challenging (4). The approval of blinatumomab, the first-in-class CD19xCD3 bispecific antibody (bsAb), significantly improved outcomes for R/R B-ALL (5). Despite being effective, several challenges remain pertinent upon blinatumomab therapy, such as the cumbersome application process and the occurrence of severe adverse reactions such as cytokine release syndrome (CRS) and immune-effector cell associated neurotoxicity syndrome (ICANS) (6).

Although T cell-based immunotherapies show high initial response rates, durable remissions are achieved in only a subset of patients (2, 7, 8). Panclonal T cell activation as achieved with classical bsAb directed to CD3 may compromise long-term efficacy by promoting immunosuppressive subsets (9). Furthermore, a subset-specific T cell activation holds promise to reduce the incidence of CRS, thus broadening patient eligibility for bsAb therapy.

Gammadelta (γδ) T cells are a subgroup of immune cells equipped with a γδ T cell receptor (TCR). While their peripheral blood levels range between 1-5% in healthy controls (10, 11), significantly higher proportions up to 20% of all lymphocytes are seen in patients after aSCT (12). In several diseases, higher abundance of γδ T cells after aSCT is associated with increased overall survival and reduced relapse mortality. Furthermore, γδ T cells do not contribute to graft-versus-host disease after aSCT (13, 14). These features suggest that γδ T cells are suitable effector cells to be stimulated by immunotherapeutic approaches (15–17). In contrast to αβ T cells, activation of γδ T cells is not HLA-restricted, which allows for off-the-shelf cell-based cellular therapy (18, 19). In a phase II trial of B-ALL, higher abundance of γδ T cells in CAR T cell products correlated with response (20). Furthermore, cytokine release upon activation of γδ T cells is lower if compared to αβ T cells (21–23), thus suggesting potentially lower CRS rates with preserved efficacy, as cytokine release is known to be dispensable for cytotoxicity (24). The most abundant γδ T cell subset in the peripheral blood are Vγ9Vδ2 T cells. They can be selectively recruited and activated to target tumor antigens. Vγ9Vδ2 T cells contribute to graft-versus-leukemia effects without promoting GVHD, likely due to their MHC-independent recognition and cytotoxicity against malignant cells (14, 25). Therapeutic strategies include adoptive transfer of ex vivo-expanded Vγ9Vδ2 T cells, *in vivo* activation with phosphoantigens or aminobisphosphonates, and combination approaches with immune checkpoint inhibitors or CAR engineering (26–28).

Few bsAb utilizing γδ T cells have been developed so far. Preclinical studies proved the efficacy and safety of γδ-targeting bsAb for the treatment of acute myeloid leukemia, chronic lymphocytic leukemia, multiple myeloma, ovarian carcinoma, and pancreatic carcinoma (22, 23, 29–32). No γδ-directed bsAb for CD19^+^ B cell malignancies has been introduced so far. However, a BTN2A1/3A1-Fc-CD19scFv fusion protein was capable of inducting lysis of CD19^+^ lymphoma cells (33). We here report on the generation and *in vitro* and ex vivo characterization of a novel CD19xγδ bsAb for the treatment of B-ALL.

## 2 Methods

### 2.1 Cell lines and patient samples

Heparinized blood was obtained from healthy male and female donors (approved by the ethics committee of the University Hospital Tübingen, authorization 156/2012BO1) and transported at room temperature. Peripheral blood mononuclear cells (PBMCs) were isolated through density gradient centrifugation using Biocoll Cell Separation Solution (Biochrom, Berlin, Germany). The average processing time was 120 minutes, with a range of 90 to 150 minutes. PBMCs were kept in RPMI 1640 medium at 37 °C and 5% CO2 and used within 24 hours. No frozen PBMCs were utilized. Cell viability, assessed using Trypan Blue and Turk’s solution (both from Sigma-Aldrich), was greater than 95%.Cell lines.

The cell lines Nalm-6 and Nalm-16 were obtained from the German Collection of Microorganisms and Cell Cultures (DSMZ, Braunschweig, Germany). CD19-transfected MCF-7 cells were a kind gift from Peter Lang, Rupert Handgretinger and Ursula Seidel (University Children’s Hospital Tuebingen) (27). Stable CD19 expression in the transfected MCF-7 cells was confirmed throughout the culture period. The medium of selected clones was supplemented with 1 mg/ml G418-BC sulfate (Biochrom). The FreeStyle™ CHO-S cells and in ExpiCHO-S™ cells, derived from Chinese hamster ovary cells, were cultured in FreeStyle™ CHO Expression Medium or ExpiCHO-S™ Expression Medium (Life Technologies, Darmstadt, Germany), supplemented with 8 mM l-glutamine (Lonza).

All cell lines were routinely tested negative for mycoplasma. PBMCs and non-transfected cell lines were cultured in RPMI 1640 medium, GlutaMAX Supplement (Life Technologies, Darmstadt, Germany) supplemented with 10% heat-inactivated fetal calf serum (PAN-biotech, Aidenbach, Germany) 100 U/ml penicillin (Sigma-Aldrich, St. Louis, USA) and 100 μg/ml streptomycin (Sigma-Aldrich), at 37 °C with 5% CO2. The studies were carried out in a laboratory following exploratory research principles, using standard protocols and conducting general research assays. The T cell assays were performed in compliance with the MIATA recommendations for reporting such methods.

### 2.2 Expansion of γδ T cells

PBMCs were plated at a density of 2 × 10^6 per well in 24-well plates and cultured in supplemented RPMI containing 100 IU/ml recombinant human IL-2 (rhIL-2) (Novartis, Basel, Switzerland) and 400 nM zoledronate (Hexal, Holzkirchen, Germany). Every 2–3 days, fresh medium containing 100 IU/ml rhIL-2 and 400 nM zoledronate was added. This resulted in cultures containing ~60% γδ T cells on days 12-15 (**Supplementary Figure 1**). After 12–15 days of culture, expanded populations were negatively selected using the magnetic activated cell sorting (MACS) TCRγ/δ + T Cell Isolation kit according to the manufacturer’s protocol (Miltenyi Biotec, Bergisch Gladbach, Germany). The purity of the isolated populations was assessed by flow cytometry and was >90% after MACS separation, and the isolated cells were then used for functional assays.

### 2.3 Hybridoma and recombinant antibody technology

The novel IgG1 antibodies clones #1-#7 were produced by immunizing female BALB/c mice with 100µg of a hVγ9Vδ2- mIgG2a protein intraperitoneally (Days 0,11, and 31) and intravenously (Day 42). Hybridoma generation was performed as described by Koehler and Milstein (28). The variable regions of the heavy (VDJ) and light (VJ) chains were sequenced by Aldevron GmbH (Freiburg, Germany).

The variable domains of the humanized anti-CD20 antibody clone 2H7 (EP2920210B1), the anti-CD19 antibody clone 4G7 (GenBank no.: AJ555479 and AJ555622), the anti-PSMA antibody clone J591 (GenBank no. FR853148.1 and FR853149.1) and the MOPC-21 antibody (GenBank no.: AAD15290.1 and AAA39002.1) were codon-optimized using the GeneArt GeneOptimizer tool for CHO cell transfection (Thermo Fisher Scientific). VH, VL, and scFv sequences were synthesized *de novo* by GeneArt (Thermo Fisher Scientific). BsAb in the Fragment antigen binding single chain (Fabsc) format were generated as previously described (29). For bsAb generation in the Immunoglobulin G single chain (IgGsc) format, a scFv fragment of the humanized anti-Vd1 antibody was inserted at the C-terminus of the IgG sequence of 2H7, 4G7, J591 or MOPC-21. The VL and VH domains were linked by a flexible (GGGGS)_3_ linker. The IgGsc construct is based on the format initially described by Coloma and Morrison (30), with modifications in the CH2 domain that include amino acid substitutions and deletions: E233P, L234V, L235A, ΔG236, D265G, A327Q, and A330S (EU index), which eliminate FcR binding and complement fixation. Extensive *in vitro* and *in vivo* characterization of the IgGsc format was performed by Zekri et al. (31).

### 2.4 Antibody production, purification and quality control

Antibodies in the IgGsc and Fabsc format as well as the hVγ9Vδ2- mIgG2a fusion protein were produced in ExpiCHO-S™ or FreeStyle™ CHO-S cells following the manufacturer’s protocol (Thermo Fisher Scientific). The antibodies were then purified from the cell culture supernatant using HiTrap^®^ MabSelect™ SuRe^®^ affinity columns and further processed by analytical and preparative size exclusion chromatography (SEC) using a Superdex 200 Increase 10/300 GL (GE Healthcare) and a HiLoad^®^ 16/600 Superdex^®^ 200 pg column (GE Healthcare), respectively. Only fractions containing the monomeric form were selected for use.

For SDS-PAGE analysis, 3–10 µg of protein was combined with 2× Laemmli Sample Buffer (Bio-Rad Laboratories) and β-mercaptoethanol, following the manufacturer’s instructions to reduce the samples. The mixtures were then heated at 95 °C for 5 minutes. Protein separation was carried out using a Mini-PROTEAN^®^ Tetra Vertical Electrophoresis Cell at a constant voltage of 100 V for 80 minutes, with Tris/glycine/SDS buffer (Bio-Rad Laboratories) as the running buffer. After separation, protein bands were stained for 1 hour with Brilliant Blue staining solution (50% H2O, 40% methanol, 10% acetic acid, and 0.1% Brilliant Blue R 250), then destained twice with Coomassie destaining solution (50% H2O, 40% methanol, and 10% acetic acid) for 1 hour and overnight, respectively.

### 2.5 Flow cytometry

Flow cytometry was employed to analyze the binding of mice serum, murine monoclonal antibodies, and bsAb to CD19^+^ Nalm-16 cells and γδ T cells. For the detection of unconjugated antibodies, PE-conjugated goat-anti-human F(ab)_2_ fragments or PE-conjugated goat-anti-mouse F(ab)_2_ fragments (Jackson ImmunoResearch) were utilized.

For flow cytometry-based assays, 100,000 CD19^+^ Nalm-16 or Nalm-6 cells were incubated in 96-well plates with 25,000 to 400,000 PBMCs or expanded γδ T cells and bispecific antibody (bsAb at 1 nm, if not indicated otherwise). Internal controls included PBMCs alone, target cells with PBMCs, and PBMCs with phytohemagglutinin L (PHA, 10 µg/ml). Flow cytometric analysis was performed after 3 days. Fc receptor binding of antibodies was blocked using Flebogamma DIF (Grifols, Barcelona, Spain) at 50 μg/ml. Absolute cell counts were obtained by acquiring equal amounts of compensation particles (Negative control vial, BD Biosciences) for each sample, enabling the calculation of the absolute level of tumor cell lysis as described before (34). For all experiments, at least 100,000 events were recorded per sample. Data acquisition was done using a FACSCanto II or FACS Celesta for functional assays or a FACSCalibur for binding analyses (BD Biosciences). Flow cytometry data were analyzed with FlowJo_V10 (Tree Star, Ashland, OR). Conjugated antibodies and the respective isotype control antibodies were obtained from BioLegend and are summarized in **Supplementary Table 1**.

### 2.6 Real-time cytotoxicity assay (xCELLigence assay)

The cytolytic activity of the expanded and isolated γδ T cells was assessed using a real-time cytotoxicity assay with an xCELLigence RTCA SP instrument (ACEA Biosciences, San Diego, CA, USA). In each well, 5 × 10^3 MCF-7-CD19 cells were seeded. After 20 hours, expanded γδ T cells (10^3 to 2.5 x 10^4) and bsAb at 10nM were added. Cell viability was recorded every 15 minutes for 48 hours. Cell indexes (CIs) were normalized to the CI at the time-point when γδ T cells were introduced, and specific lysis was calculated relative to control wells that lacked any effector γδ T cells. The Kill-Time-50 (KT50) was defined as the time taken after PBMC addition for 50% of the adherent tumor cells to be eradicated.

### 2.7 Statistical analysis

Data are presented as means ± SD or SEM, as specified in the figure legends. Boxplots with Tukey whiskers or min/max whiskers were used. Group means of continuous variables were compared using the two-sided t-test or ANOVA. Median values of nonnormally distributed variables were compared using the Mann-Whitney U test or Wilcoxon test. Statistical significance was determined using GraphPad Prism version 9.4 (GraphPad Software, San Diego, CA, USA). A p-value <.05 was considered statistically significant.

## 3 Results

### 3.1 Generation of novel monoclonal Vγ9Vδ2 antibodies

**Figure 1:**
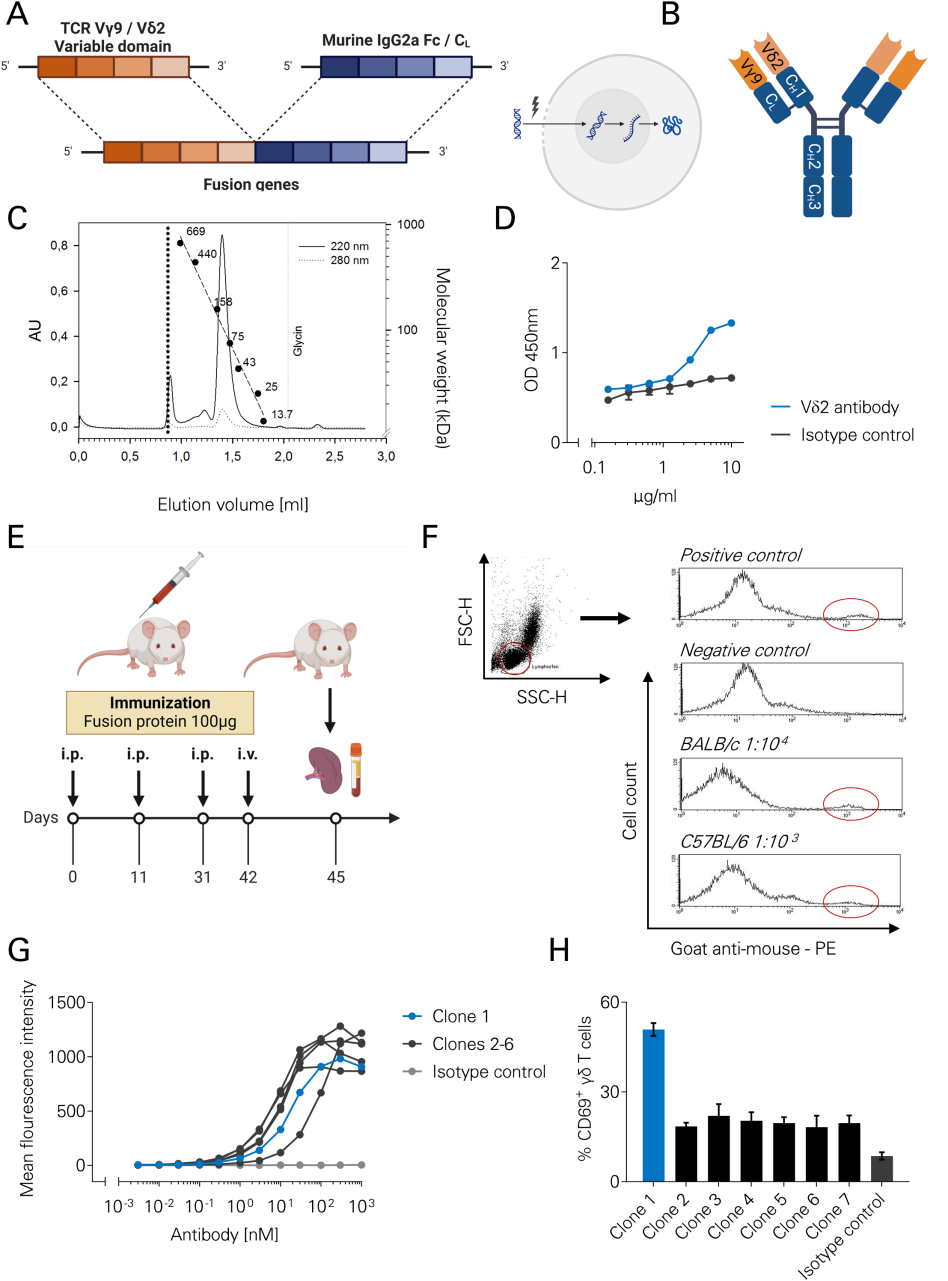
Generation of a novel TCRγδ binding antibody using hybridoma technology. **(A)** Schematic representation of the cloning strategy. The construct includes murine IgG2a CH1–CH3 domains fused to the human TCR Vγ9 domain, and a murine CL domain fused to the human TCR Vδ2 domain. **(B)** Schematic of the resulting human Vγ9Vδ2–murine IgG2a (hVγ9Vδ2–mIgG2a) fusion protein. **(C)** Analytical size-exclusion chromatography (gel filtration) of the fusion protein (10 µg), produced in Sp2/0 cells and purified using protein A affinity chromatography. **(D)** ELISA detecting the fusion protein using a goat anti-human Fcγ antibody (5 µg/mL) as the capture antibody, increasing concentrations of the hVγ9Vδ2–mIgG2a fusion protein, and a biotin-labeled anti-Vδ2 detection antibody. Detection was performed using a streptavidin–HRP system. **(E)** Immunization schedule for mice injected with the hVγ9Vδ2–mIgG2a fusion protein. **(F)** Flow cytometric analysis of serum samples from immunized mice on day 36. Specific binding of murine anti-hVγ9Vδ2 antibodies to human peripheral blood mononuclear cells (PBMCs) was detected using a goat anti-mouse secondary antibody. BALB/c serum was diluted 1:10,000 and C57BL/6 serum 1:1,000. A commercial murine anti-TCRγδ antibody was used as an isotype control. **(G)** Binding of murine monoclonal anti-Vγ9Vδ2 antibodies to isolated human γδ T cells was assessed by flow cytometry following incubation with increasing antibody concentrations and staining with a PE-conjugated anti-mouse secondary antibody. The isotype control mF19 was included. **(H)** Human PBMCs (n=3) were incubated for 96 hours with 5 nM of the indicated monoclonal murine anti-Vγ9Vδ2 antibodies. CD69 expression on γδ T cells was measured by flow cytometry as a surrogate marker for T cell activation.

Gammadelta T cells are potent immune effector cells, which may be activated by specific antibodies. To eventually create a novel CD19/CD20xγδ bsAb, a panel of monoclonal antibodies (mAbs) capable of activating γδ T cells were generated. To this end, a fusion protein comprised of the Vγ9Vδ2 variable domains, and a murine IgG2a Fc/CL domain was cloned (**Figure 1A**). The resulting h(uman) Vγ9Vδ2- m(urine) IgG2a fusion protein is depicted in **Figure 1B**. Analytical gel filtration showed a peak at the expected molecular weight of ~150 kDa with low aggregation (**Figure 1C**). An ELISA assay using a goat anti-human Fcγ antibody and a biotin-labelled anti-Vδ2 antibody proved the presence of an intact Vδ2 domain within the fusion protein (**Figure 1D**). To generate murine monoclonal antibodies, mice were immunized with 100µg of the hVγ9Vδ2- mIgG2a protein intraperitoneally (Days 0,11,31), followed by intravenous application on day 42 (**Figure 1E**). A flow cytometric binding analysis using mice serum on day 36 showed specific binding of murine anti-hVγ9Vδ2 antibodies on human peripheral blood mononuclear cells (PBMC). Since BALB/c rather than C57BL/6 mice showed a robust antibody induction, only BALB/c spleen cells were subjected to hybridoma technology (**Figure 1F**). To this end, on day 45, spleens were collected and fused according to the protocol by Koehler and Milstein (28).

### 3.2 Characterization of the different Vγ9Vδ2 antibodies

Following isolation, six different antibody producing hybridomas were generated (**Supplementary Figures 2A, B**). All the respective mAbs bound to isolated human γδ T cells with high affinity (EC50 ~ 10nM, **Figure 1G**). The specificity of the clones was assessed by Christian Welker and Karin Schilbach (Children’s Hospital, University Hospital Tübingen, Germany) using flow cytometric binding analysis on a human Vγ9Vδ1 T cell clone. Clone #1 was found to bind to Vγ9, whereas #2–7 were Vδ2-specific (**Supplementary Figures 2C, D**). To evaluate activating properties, the mAbs were incubated with human PBMC. As a surrogate marker for T cell activation, CD69 expression on human γδ T cells was determined by flow cytometry. CD69 expression was >50% in T cells treated with clone #1 compared to < 30% in T cells treated with the other clones (#2-7, **Figure 1H**). Accordingly, clone #1 binding to Vγ9 was chosen for further development of the Vγ9Vδ2 bsAbs.

### 3.3 Generation of recombinant bsAbs targeting TCRgammadelta

**Figure 2:**
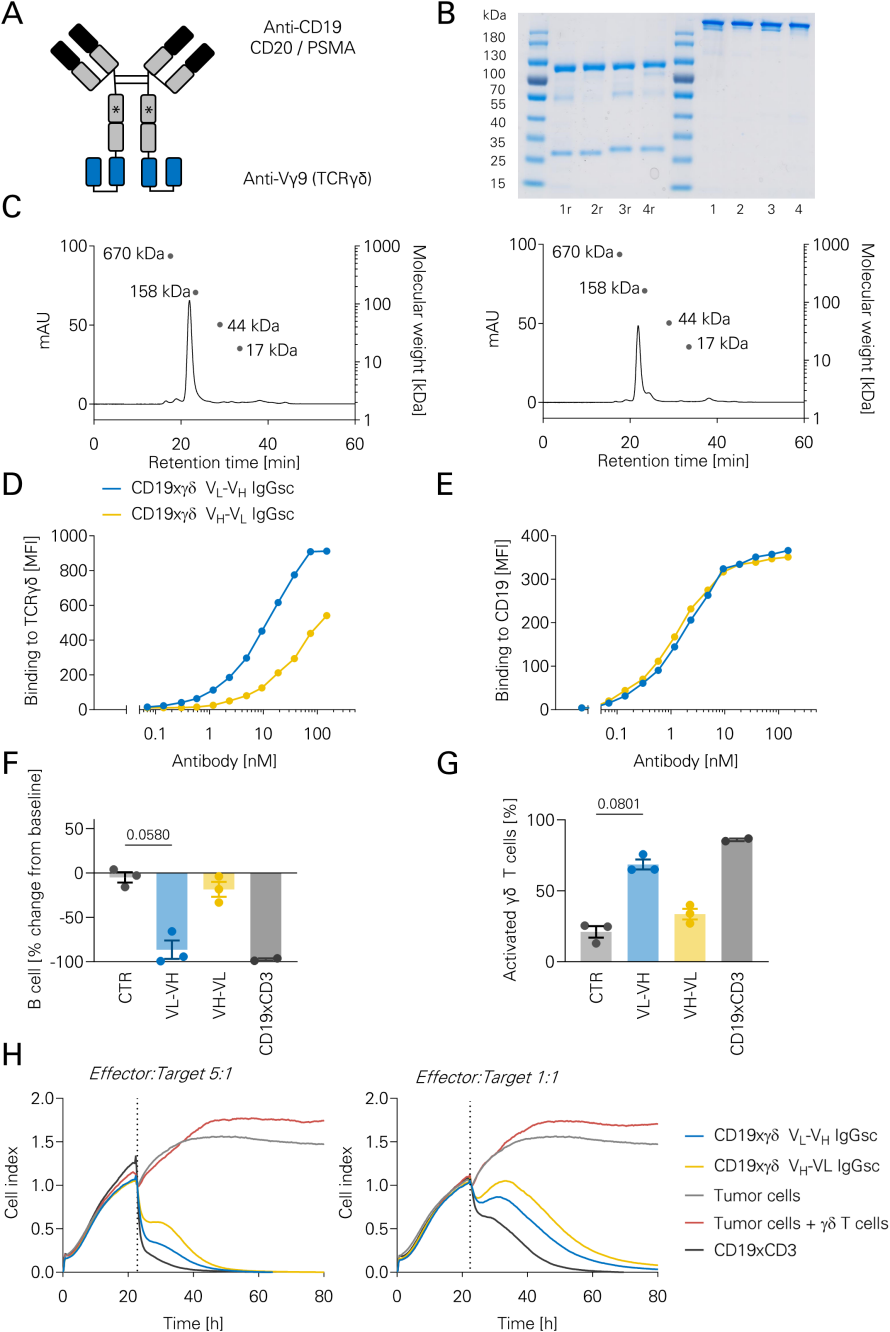
Production and characterization of novel bispecific CD19xTCRγδ antibodies. **(A)** Schematic representation of anti-CD19/CD20/PSMAxTCRγδ bispecific antibodies (bsAbs) in the IgGsc format. Asterisks indicate CH2 domain silencing to prevent Fcγ receptor binding. **(B)** SDS-PAGE analysis of four different bsAb (1, CD19xγδ VL-VH; 2, PSMAxγδ VL-VH; 3, CD19xγδ VH-VL; 4, PSMAxγδ VH-VL). Lanes labeled ‘r’ indicate reducing conditions. An uncropped image of the gel is shown as **Supplementary Figure 9B**. **(C)** Size-exclusion chromatography profiles of CD19xγδ bsAbs in VH-VL (left) and VL-VH (right) orientations. **(D)** Binding of the indicated bsAbs to human TCRγδ was assessed by flow cytometry. Isolated γδ T cells were incubated with increasing bsAb concentrations, followed by detection with goat anti-human PE-conjugated secondary antibody. Data are shown as mean fluorescence intensity (MFI). Representative data from one out of three experiments with different donors. **(E)** Binding of bsAbs to CD19 was evaluated using the CD19^+^ acute lymphoblastic leukemia (ALL) cell line Nalm-16. Cells were incubated with increasing bsAb concentrations and stained with goat anti-human PE-conjugated secondary antibody for flow cytometry analysis. Representative data from one out of three experiments with different donors. **(F)** Functional activity of bsAbs in a B cell depletion assay. Human PBMCs and autologous γδ T cells (5:1 ratio) from healthy donors (n=3) were incubated for 48 hours with the indicated bsAbs (CD19xγδ VH-VL, CD19xγδ VL-VH, CD19xCD3, and PSMAxγδ VH-VL as isotype control). B cell depletion was measured by flow cytometry. Data are shown as individual values and bar graphs; statistical analysis was performed using the Kruskal–Wallis test followed by Dunn’s multiple comparisons. **(G)** T cell activation assessed by CD69 expression on γδ T cells after 48-hour incubation with the indicated bsAbs, as described in **(F)**. Flow cytometry was used to determine CD69 levels. Data represent individual values (n=3) and bar graphs; Kruskal–Wallis test with Dunn’s *post hoc* analysis **(H)** Cytotoxicity assay using the xCELLigence real-time cell analysis system. CD19-transfected MCF-7 cells were seeded and allowed to adhere for 24 hours, followed by co-culture with γδ T cells at effector: target (E:T) ratios of 5:1 and 1:1 in the presence of 10 nM bsAb. Tumor cell viability was monitored every 15 minutes based on impedance (cell index).

Next, bsAbs in our recombinant IgGsc format were generated as previously described (31, 32). To this end, two single chain fragment variables (scFv) derived from the hybridoma-derived Vγ9 specific murine antibody clone #1 were generated: VL-VH orientation versus VH-VL orientation. Fab sites binding to CD19, CD20 or PSMA (as isotype control for leukemia/lymphoma assays) were used (**Figure 2A**). Antibodies were generated by transient transfections as described in the Methods section. SDS-PAGE showed bands at the expected molecular weight (**Figure 2B**). Size exclusion chromatography showed sufficient production and low rates of aggregates of the generated IgGsc molecules (**Figure 2C**). To evaluate the binding affinity of the molecules, flow cytometric binding analyses were performed. The IgGsc molecule with the scFV in VL-VH orientation showed a higher binding affinity to the TCRγδ receptor (**Figure 2D**). Binding to the tumor-associated antigen (TAA) CD19 was similar with both molecules (**Figure 2E**). The higher binding affinity of the VL-VH molecule translated into a more potent B cell depletion as assessed by a flow cytometric depletion assay with PBMC (**Figure 2F**). Likewise, VL-VH induced a more robust γδ T cell activation mirrored by higher CD69 expression in a flow cytometric depletion assay comparable to that achieved with a CD19xCD3 bsAb as positive control (**Figure 2G**). Dose-dependent killing was not compared between both molecules. To validate these findings, a real-time impedance-based kill assay with expanded γδ T cells and CD19 transfected adherent MCF-7 cells was performed. While both molecules showed anti-tumor activity, a more potent reduction of tumor cells was observed with CD19xγδ VL-VH versus VH-VL (50% reduction after 24.6 hours versus 33.6 hours, **Figure 2H**; **Supplementary Figure 3**). Due to the superior activity, CD19xγδ VL-VH was selected. Constructs in both our bivalent IgG-based format IgGsc and the monovalent Fab-based Fabsc format were generated. Bispecific antibodies in both formats showed comparable efficacy (**Supplementary Figures 4**, **S5**). Due to increased incidence of aggregates (35), IgGsc bsAb rather than Fabsc bsAb were chosen for further development. To target CD19 and CD20, bsAbs directed against both antigens were generated.

### 3.4 Lysis of autologous B cells by T cells stimulated with TCRgammadelta bsAb

**Figure 3:**
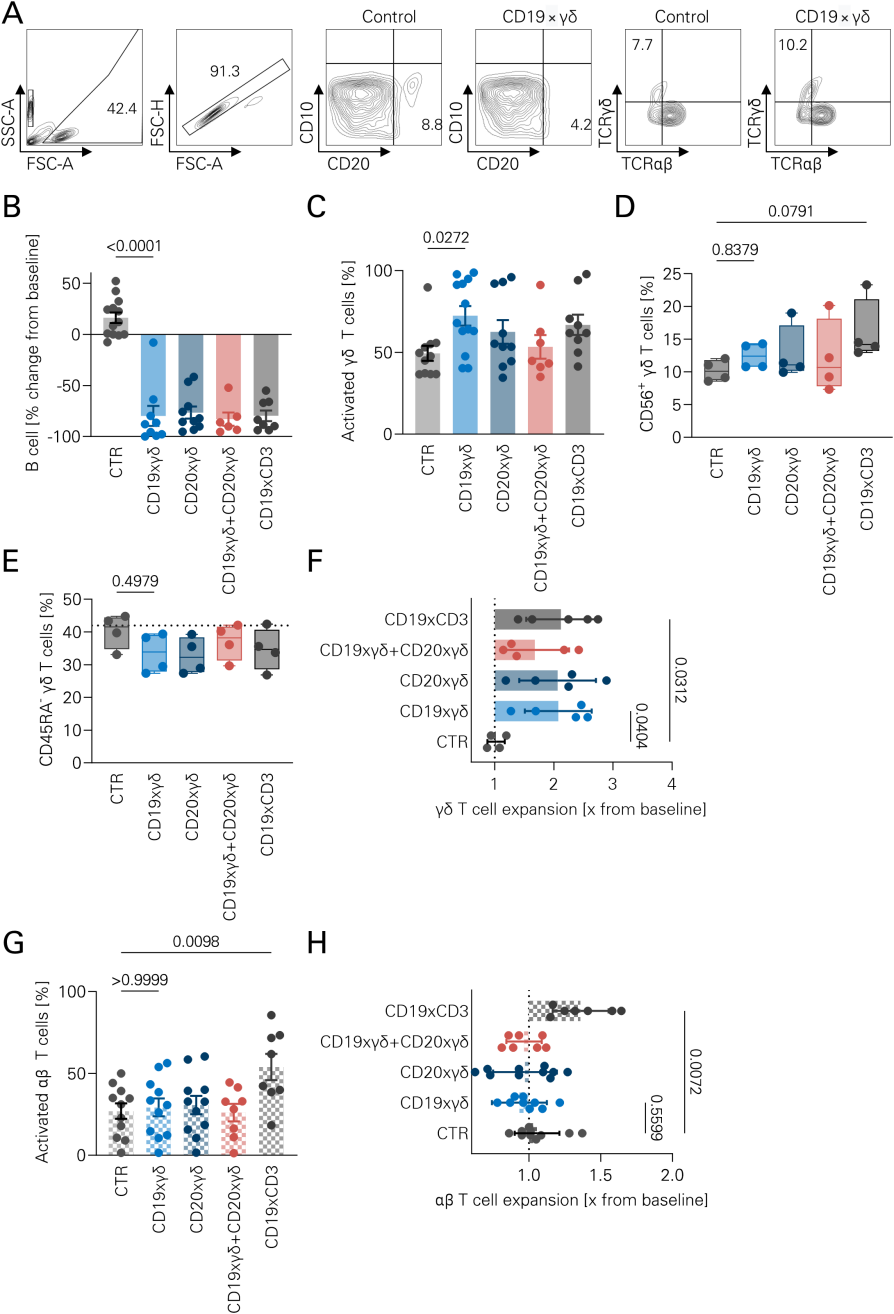
Lysis of autologous B cells by γδ T cells stimulated with CD19xγδ and CD20xγδ bsAb. **(A)** Representative gating strategy used for flow cytometric analysis. Gates include total cells (FSC/SSC), singlets (FSC-A/FSC-H), B cells (CD10^-^CD19^+^), γδ T cells (TCRγδ^+^TCRαβ^-^), and additional markers including CD56, CD69, and CD45RA (not shown). **(B)** Human PBMCs were cultured with the indicated bsAbs for 72 hours. Absolute B cell numbers were quantified by flow cytometry. A MOPCxγδ bsAb was used as a negative control (CTR). Data are presented as individual values with bar graphs (n=7); statistical analysis was performed using one-way ANOVA with Bonferroni correction. **(C)** CD69 expression was measured as a surrogate marker of T cell activation following 72-hour PBMC culture with the indicated bsAbs. Flow cytometric analysis was performed. Data represent individual values (n=10) with bar graphs, statistical analysis by one-way ANOVA with Bonferroni correction. **(D)** CD56 expression on γδ T cells was evaluated after 72-hour incubation with different bsAbs. Data shown as individual values (n=4), presented in box plots with min/max whiskers. Statistical analysis: Kruskal–Wallis test with Dunn’s multiple comparisons. **(E)** CD45RA expression on γδ T cells was assessed by flow cytometry following 72-hour incubation with bsAbs (5 nM). Data (n=4) are shown as box plots with min/max whiskers; Kruskal–Wallis test with Dunn’s *post hoc* test. **(F)** γδ T cell expansion was measured after 72-hour PBMC culture with bsAbs (5 nM). Expansion is expressed as the fold increase in absolute γδ T cell numbers relative to untreated control PBMCs. Data shown as individual values (n=5) with bar graphs; analyzed by one-way ANOVA with Bonferroni correction. **(G)** CD69 expression on αβ T cells was used as a surrogate marker for activation following 72-hour incubation with the indicated bsAbs. Flow cytometric data (n=8–11) are presented as individual values with bar graphs, ANOVA with Bonferroni correction. **(H)** αβ T cell expansion was quantified after 72-hour incubation with 5 nM bsAbs. Fold expansion was calculated as the ratio treated to untreated PBMC αβ T cell counts. Data shown as individual values (n=7) with bar graphs; statistical analysis via ANOVA with Bonferroni correction. Panels **A**, **B** and **D** were created in BioRender.

To further characterize our bsAb constructs, preactivated allogeneic γδ T cells were cultured with healthy B cells (**Supplementary Figures 6A, B**). A dose-dependent B cell depletion was observed (**Supplementary Figure 6C**), also at unfavorable effector: target (E:T) ratios (**Supplementary Figure 6D**). Effects were clearly dependent on dose and E:T ratios, which prompted us to study B cell depletion ex vivo. Freshly isolated PBMC containing a small proportion of Vγ9Vδ2 T cells were incubated with CD20xγδ and CD19xγδ bsAb. The flow cytometric gating strategy is shown in **Figure 3A**. Both CD19xγδ, CD20xγδ, and their combination were capable of subtotal reduction of B cells in this setting (**Figure 3B**). BsAbs induced CD69 expression, thus suggesting activation of the respective Vγ9Vδ2 subset (**Figure 3C**). No significant induction of CD56 expression (**Figure 3D**) or differentiation, as mirrored by the rate of CD45RO^+^ γδ T cells, was observed (**Figure 3E**). Antibody treatment induced approximately 2-fold γδ T cell expansion within 72 hours (**Figure 3F**). Furthermore, neither the CD19 nor the CD20 directed bsAb led to activation or expansion of αβ T cells, in contrast to the positive control blinatumomab (**Figures 3G, H**). No synergistic effects were seen upon combined application of both, the CD19xγδ and CD20xγδ bsAb.

### 3.5 Lysis of leukemic cell lines by T cells and TCRgammadelta bsAbs

**Figure 4:**
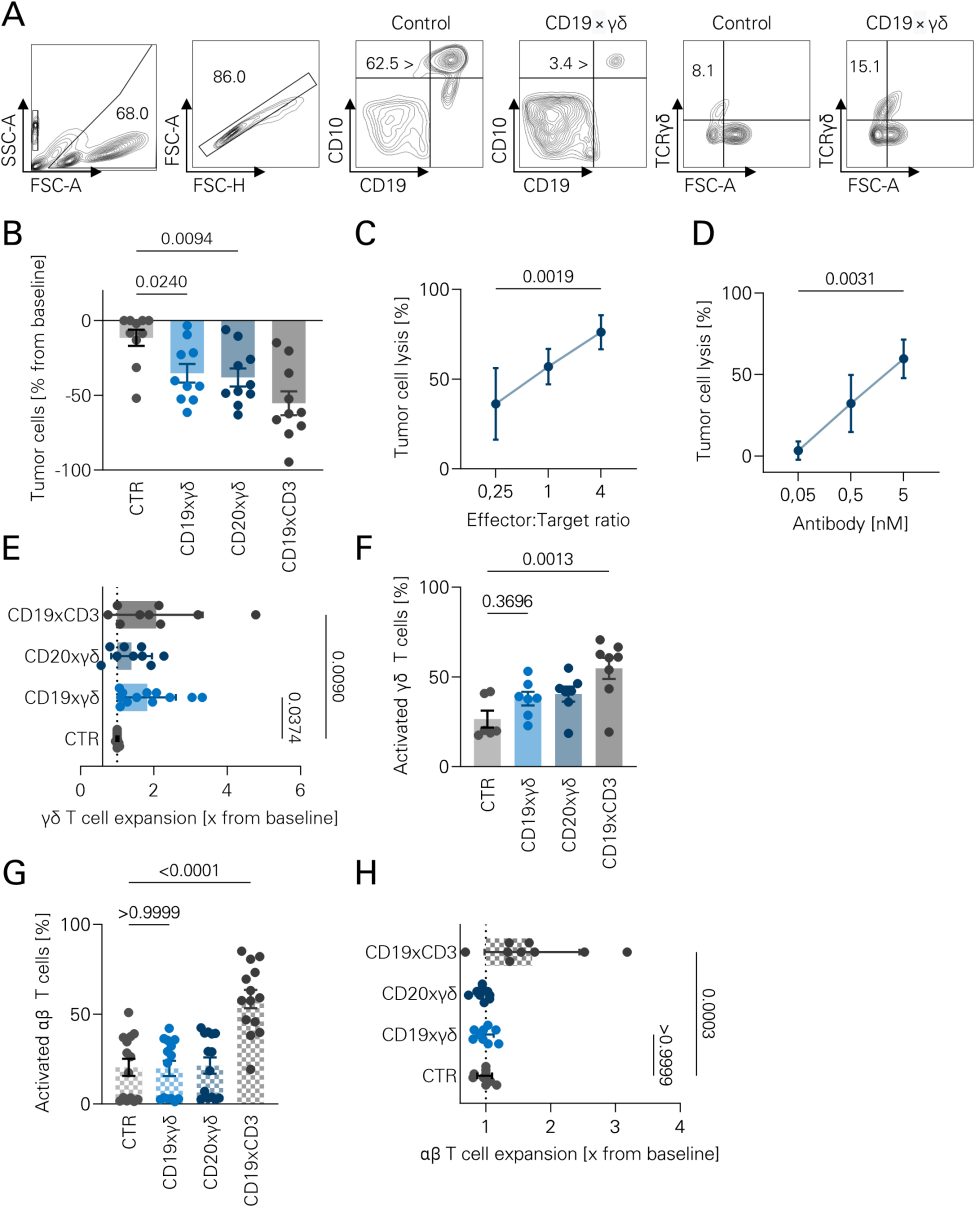
Subset-restricted activation of γδ T cells against leukemic cell lines. **(A)** Representative gating strategy used for flow cytometric analysis. Gated populations include: total cells (FSC/SSC), singlets (FSC-A/FSC-H), B cells (CD10^-^CD19^+^), Nalm-16 leukemic cells (CD10^+^CD19^+^), γδ T cells (TCRγδ^+^TCRαβ^-^), and activated cells (CD69^+^; not shown). **(B)** Isolated γδ T cells and Nalm-16 cells were co-cultured at an effector-to-target (E:T) ratio of 1:1 in the presence of the indicated bsAbs for 72 hours. Absolute tumor cell numbers were quantified by flow cytometry. A MOPCxγδ bsAb served as a negative control (CTR). Data represent individual values (n=10), shown as bar graphs; statistical analysis performed using one-way ANOVA with Bonferroni correction. **(C)** Flow cytometric lysis assays were conducted as in **(B)**, using CD20xγδ bsAb (5 nM) and varying E:T ratios. Tumor cell lysis was analyzed by flow cytometry. Data are shown as individual values (n=6), ANOVA with Bonferroni correction. **(D)** Dose-response flow cytometric lysis assays were performed at a fixed E:T ratio of 1:1 using increasing concentrations of CD20xγδ bsAb. Data represent individual values (n=3); statistical analysis by Kruskal–Wallis test with Dunn’s multiple comparisons. **(E)** Human PBMCs were incubated with different bsAbs (5 nM) for 72 hours. γδ T cell expansion was calculated as the fold change in absolute cell numbers relative to untreated controls. Data are shown as individual values (n=9), bar graphs; statistical analysis using ANOVA with Bonferroni correction. **(F)** CD69 expression on γδ T cells was assessed by flow cytometry as a marker of activation following 72-hour incubation with the indicated bsAbs. Data are shown as individual values (n=7–8), bar graphs; statistical analysis via ANOVA with Bonferroni correction. **(G)** CD69 expression on αβ T cells was evaluated after 72-hour incubation with different bsAbs to assess off-target activation. Data (n=9) are presented as individual values, bar graphs; statistical analysis using ANOVA with Bonferroni correction. **(H)** αβ T cell expansion was measured after 72-hour PBMC incubation with 5 nM bsAbs. Expansion was expressed as fold change in absolute αβ T cell numbers relative to untreated PBMCs. Data represent individual values (n=14), shown as bar graphs, ANOVA with Bonferroni correction.

Next, leukemic cell lines were incubated with expanded γδ T cells and the different bsAbs. The gating strategy for flow cytometric assays with the B-ALL cell line Nalm-6 is depicted in **Figure 4A**. Both CD19xγδ and CD20xγδ bsAb could induce leukemia cell lysis, albeit to a lesser extent when compared to blinatumomab (**Figure 4B**). Effects were dependent on E:T ratio. An E:T ratio of 4:1 resulted in 76% tumor cell lysis, whereas a ratio of 1:4 achieved 36% tumor cell lysis (**Figure 4C**). Furthermore, tumor cell killing occurred in a dose-dependent manner (**Figure 4D**). Within 72 hours, γδ T cells expanded 2-fold upon treatment with both bsAb (**Figure 4E**). The bsAbs both increased T cell activation, albeit to a lesser extent compared to blinatumomab (**Figure 4F**). Subset-specific T cell activation was confirmed as the bsAb did not induce activation or expansion of αβ T cells (**Figures 4G, H**). Results were confirmed in a second B-ALL cell line, namely Nalm-16. Equivalent results regarding T cell activation, proliferation and tumor cell lysis were observed (**Supplementary Figures 7**, **8**).

### 3.6 Ex vivo killing of B-ALL blasts by bsAb-activated Vγ9Vδ2 T cells

**Figure 5:**
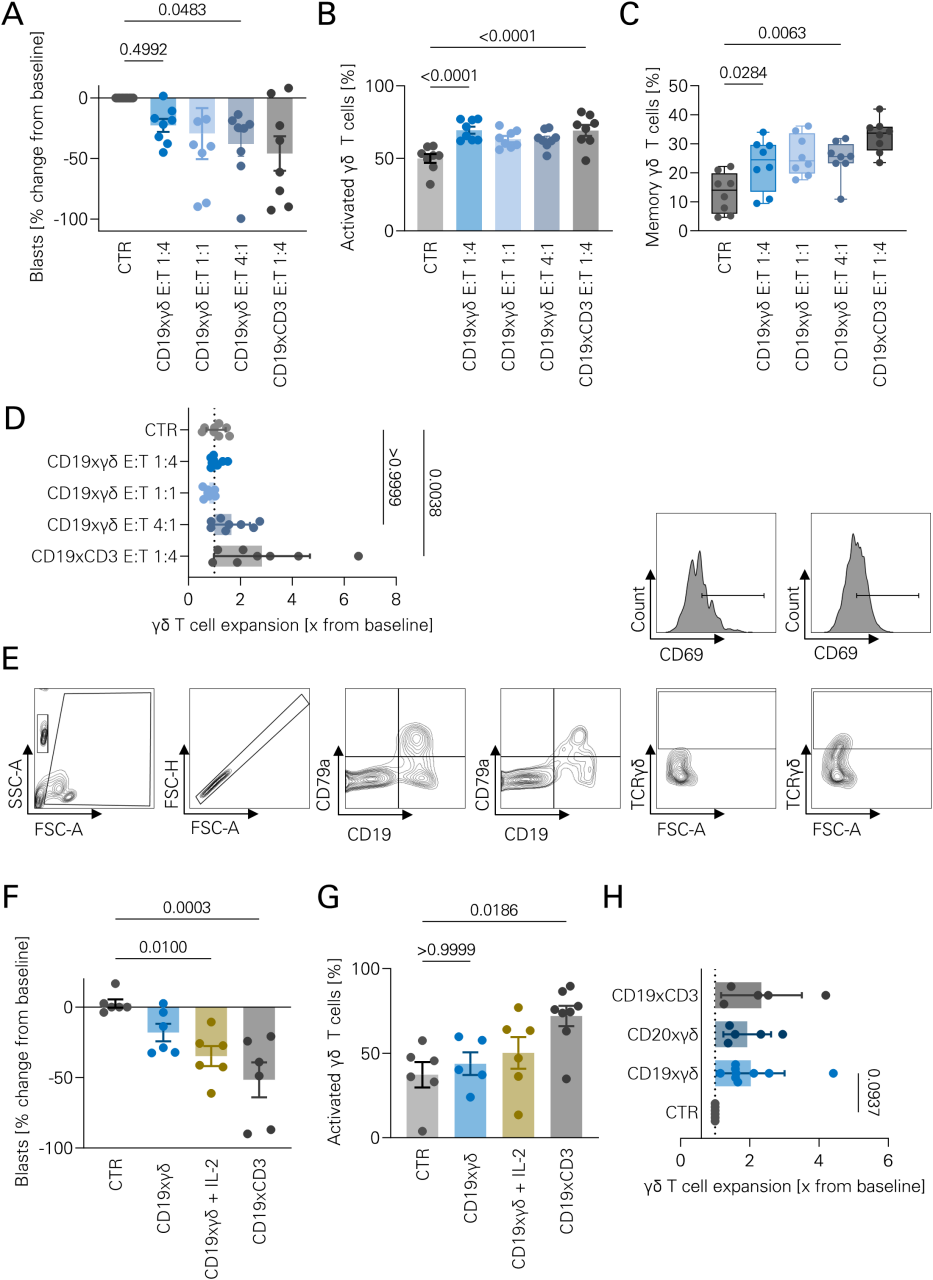
Ex vivo lysis of primary leukemic cells by γδ T cells mediated by bsAbs. **(A)** Isolated γδ T cells and PBMCs containing >50% leukemic blasts were co-cultured at the indicated effector-to-target (E:T) ratios in the presence of the indicated bsAbs for 96 hours. Absolute tumor cell numbers were quantified by flow cytometry. A MOPCxγδ bsAb at an E:T ratio of 1:1 served as a negative control (CTR). Data represent individual values (n=8), shown as bar graphs, statistical analysis by one-way ANOVA with Bonferroni correction. **(B)** CD69 expression on γδ T cells was measured by flow cytometry after 96-hour co-culture with leukemic blasts and the indicated bsAbs. CD69 served as a surrogate marker of T cell activation. Data shown as individual values (n=8), bar graphs; ANOVA with Bonferroni correction. **(C)** CD45RA expression on γδ T cells was assessed following co-culture as in **(A)**. The percentage of CD45RA^-^ cells was used as a surrogate marker for memory T cell phenotype. Data (n=8) shown as individual values; Kruskal–Wallis test with Dunn’s multiple comparisons. **(D)** PBMCs containing leukemic blasts were co-cultured with isolated γδ T cells and bsAbs (5 nM) for 96 hours. γδ T cell expansion was calculated as fold change in absolute cell numbers relative to untreated PBMCs. Data shown as individual values (n=8), bar graphs; ANOVA with Bonferroni correction. **(E)** Representative gating strategy for autologous leukemic blast lysis assays. Gated populations include total cells (FSC/SSC), singlets (FSC-A/FSC-H), leukemic blasts (e.g., CD79a^+^CD19^+^), γδ T cells (TCRγδ^+^), and CD69^+^ cells (histograms shown). **(F)** PBMCs containing 10–20% leukemic blasts were cultured with the indicated bsAbs for 96 hours. Tumor cell numbers were assessed by flow cytometry. A MOPCxγδ bsAb served as a negative control (CTR). Data represent individual values (n=6), bar graphs; statistical analysis using ANOVA with Bonferroni correction. **(G)** Autologous leukemic blast lysis assays were performed as described in **(F)**. CD69 expression on γδ T cells was evaluated after 96 hours as a marker of activation. Data shown as individual values (n=6), bar graphs; ANOVA with Bonferroni correction. **(H)** γδ T cell expansion was assessed following 96-hour culture of PBMCs containing 10–20% leukemic blasts in the presence of different bsAbs (5 nM). Fold expansion was calculated relative to untreated PBMC controls. Data shown as individual values (n=5), bar graphs; ANOVA with Bonferroni correction.

To further evaluate the two bsAbs, primary leukemic blasts of ALL patients were cocultured with isolated allogeneic γδ T cells at different E:T ratios. **Supplementary Figure 8** depicts the flow cytometric gating strategy. Patient characteristics are listed in **Table 1**. Treatment at an E:T ratio of 1:4 and 4:1 induced a tumor cell reduction of 22.6% and 38.0%, respectively (**Figure 5A**). Expanded γδ T cells were further activated by the CD19xγδ bsAb (**Figure 5B**). Memory γδ T cells (defined as TCRγδ+ CD45RO^+^) were significantly increased upon treatment with the CD19xγδ bsAb (**Figure 5C**). A significant overall T cell proliferation was not observed in this setting, in contrast to ~3 fold expansion with blinatumomab (**Figure 5D**). Finally, PBMC samples of untreated ALL patients containing 10-20% leukemic blasts were cultured with the CD19xγδ bsAb. An exemplary gating strategy for a patient with CD19^+^CD79a^+^ B-ALL blasts is shown as **Figure 5E**. Significant tumor cell reduction was observed with CD19xγδ bsAb, which could be further augmented by addition of IL-2 (**Figure 5F**). Gammadelta T cells displayed a high background activation in this setting, which could be further increased by blinatumomab, but not the CD19xγδ bsAb (**Figure 5G**). However, a significant γδ T cell expansion was observed after 96 hours, with a 2-fold increase in numbers compared to control (**Figure 5H**). CD19 antigen escape is a known resistance mechanism after bsAb therapy. No changes in CD19 expression on blasts was observed after 96 hours of treatment with either bsAb.

**Table 1:** Patient characteristics.

#	Age	Sex	Diagnosis	Leuko: blast ratio	% Blasts	Immunophenotype	Time point	Material
1	25	F	c-B-ALL Brc-abl^+^	0.63	59	CD34^+^ CD10^+^ CD19^+^	FD	PB
2	76	F	c-B-ALL	0.08	91	CD34^+^ CD10^+^ CD19^+^	Relapse	PB
3	58	M	c-B-ALL	0.66	51	CD34^+^ CD10^+^ CD19^+^ CD22^+^	FD	PB
4	23	M	proB-ALL	0.78	18	CD34^+^ CD10- CD19^+^	Relapse	PB
5	22	M	proB-ALL	0.07	92	CD34^+^ CD10- CD19^+^	FD	PB
6	29	F	proB-ALL	0.02	88	CD34^+^ CD79a^+^ CD19^+^	FD	PB
7	67	F	c-B-ALL Brc-abl^+^	2.10	17	CD34^+^ CD10^+^ CD19^+^	FD	PB
8	52	F	c-B-ALL	7.66	6	CD22^+^CD79a^+^ CD19^+^	FD	PB

F, female; M, male; c-B-ALL, common B acute lymphoblastic leukemia; CD, cluster of differentiation; FD, first diagnosis; PB, peripheral blood.

In summary, the CD19xγδ bsAb generated in this work is able to activate and expand γδ T cells and induces B cell depletion and killing of B-ALL blasts.

## 4 Discussion

In this work, we report on the generation and preclinical *in vitro* and ex vivo characterizing of bsAb which specifically activate Vγ9Vδ2 T cells.

Standard treatment of B-ALL involves multiagent chemotherapy and aSCT. The introduction of blinatumomab and thus T cell-based immunotherapy revolutionized the treatment of B-ALL (5, 6). Similarly, chimeric antigen receptor (CAR) equipped T cells comprise a potentially curative treatment option for B-ALL patients (7, 8). Despite initially high response rates, only a subset of patients is cured by the available T cell-based immunotherapeutic approaches. Several studies suggest that the preexisting T cell repertoire and fitness affects long-term outcomes of those therapies (36–38). Pan T cell activation as achieved by Blinatumomab might lead to induction of immunosuppressive T cell subsets, thus hampering long-term efficacy (9, 39). Furthermore, pan T cell activation might result in excessive cytokine release leading to a potentially lethal CRS (40, 41). Both potentially can be overcome by selectively targeting cytotoxic T cells subsets that comprise only a minority of the available T cells. One approach would be the activation of Vγ9Vδ2 T cells, the beyond ab T cells most abundant subset in the peripheral blood. Lower incidence of CRS improves the safety profile of this immunotherapeutic approach and may allow for a higher number of patients to qualify for bsAb therapy (22, 23). Several groups have introduced concepts to employ Vγ9Vδ2 T cells in either adoptive cell therapy or CAR T cell therapy (42–44). In several hematological malignancies, Vγ9Vδ2 CAR T cell therapy has been evaluated preclinically alone or as combined approaches of adoptive cell therapy and bsAb therapy (45, 46). Surprisingly, no data are so far available for B-ALL, which prompted us to investigate CD19xγδ and CD20xγδ bsAbs for the treatment of this disease.

So far, only a few bsAbs targeting γδ T cells were described. In 1989, Ferrini et al. created a Mov19xTCRγδ IgG1/lgG2a bsAb, capable of lysing ovarian carcinoma cell lines (30). In 2014, Oberg et al. reported on a Her2neu (ERBB2) x Vγ9 bsAb for the treatment of pancreatic adenocarcinoma in a tribody format (29). King et al. generated EGFRxVδ2 bsAbs in different formats that successfully activated Vγ9Vδ2 T cells against EGFR-expressing solid tumors *in vitro*, *in vivo* and ex vivo (23). In the same publication, using gastrointestinal tumor samples, tumor-infiltrating Vγ9Vδ2 T cells exhibited low levels of LAG-3 and TIM-1 (23). In non-human primates, the EGFRxVδ2 bsAb induced Vγ9Vδ2 T cell activation, but not cytokine release, suggesting a low probability of CRS in Vγ9Vδ2-targeting bsAb therapy. Ganesan et al. characterized the CD123xVγ9 bsAb for the treatment of AML. In contrast to pan T cell activation, Vγ9Vδ2 T cell activation did not induce excessive cytokine release. Interestingly, the authors observed increased exhaustion marker expression upon activation, which did not interfere with cytotoxic abilities of the Vγ9Vδ2 T cells (22) In summary, different bsAbs targeting either Vγ9 or Vδ2 have been developed, suggesting that both subunits might be suitable targets for immunotherapy. In our analyses, the mAb directed against Vγ9 seemed more potent than the mAbs directed against Vδ2. However, the lack of binding to Vγ9Vδ1 T cells does not fully prove specificity against Vγ9. Further characterization should include epitope mapping, since an epitope comprised of both Vγ9 and Vδ2 is still possible. Furthermore, our bsAb is potentially able to activate the small subset of Vγ9^+^Vδ2^-^ T cells, which was not studied in detail in this work and might contribute to the seemingly higher T cell activation in our hybridoma clone of choice.

Circulating Vγ9Vδ2 T cells are mostly central memory (CD27^+^, CD45RA^-^) or effector memory (CD27^-^, CD45RA^-^) subsets. Upon activation, these cells differentiate into effector memory cells (CD27^-^, CD45RA^-^) and exhibit elevated intracellular levels of Granzyme B and Perforin (22). Yet, T cell exhaustion in Vγ9Vδ2 T cells is incompletely understood. Guo et al. observed increased TIM-3 expression on γδ T cells upon persistent stimulation, contributing to dysfunctional states. By combining TIM-3 blocking antibodies with an EpCamxCD3 bsAb, antitumor effects were enhanced (47). In case of γδ T cell exhaustion upon stimulation with our CD19xVγ9Vδ2 bsAb, checkpoint blockade could be explored in a combinatorial regimen.

While showing robust activity, this analysis has some weaknesses. Firstly, our newly developed bsAb resulted in moderate γδ T cell activation. Due to gating on pan- γδ T cells, the CD69 expression in the Vγ9^+^ subset might be higher. The magnitude of tumour reduction observed with the CD19 × γδ bsAb is more modest than that achieved with CD3-directed agents such as blinatumomab. However, this difference should be interpreted in the context of the distinct biology and therapeutic intent of Vγ9Vδ2 T-cell engagement. Also, *in vitro* and ex vivo experiments were not always performed using saturating antibody concentrations, which might have had a negative effect on tumor cell lysis in these settings. Furthermore, the use of fixed timepoints rather than dynamic monitoring of T cell activation, proliferation and killing might have led to underestimation of certain effects.

Further evaluation should involve studies on the expression of T cell exhaustion markers upon treatment with our CD19xVγ9Vδ2 bsAb. Likewise, activation thresholds that trigger T cell activation cascades should be assessed to avoid excessive or tonic activation that might compromise functional persistence or safety. Antigen density-dependent killing might be a reason for the absence of synergy with the combination of CD19x Vγ9Vδ2 and CD20x Vγ9Vδ2 bsAb. Tumor rechallenge could prove functional persistence of Vγ9Vδ2 T cells after stimulation with our bsAb.

Allogeneic stem cell transplantation is an important consolidation therapy in a variety of hematological cancers. Eligible patients with B-ALL might be subjected to aSCT (1). T cells exhibiting a graft-versus-leukemia effect are crucial for the efficacy of aSCT (48, 49). However, graft-versus-host disease may be harmful, often requiring intensified immunosuppressive therapy, eventually accounting for a substantial proportion of non-relapse mortality (50). Minculescu et al. and other groups showed that γδ T cells do not contribute to graft-versus-host disease (13, 51). Higher numbers of cytotoxic γδ T cells in the allograft correlate with improved survival (25). This suggests that γδ T cell activation is a feasible immunotherapeutic approach after aSCT, even in patients with preexisting graft-versus-host disease. Targeting of γδ T cells might therefore be an effective approach to target relapses or persistent/refractory disease without exacerbation of graft-versus-host disease.

Different modalities for the treatment of MRD positive patients with B-ALL after allo-SCT have been evaluated. The efficacy of donor lymphocyte infusion (DLI) in B-ALL patients is limited (52). Furthermore, DLI may only be applied to patients without higher grade GvHD and relies on the availability of the donor. Combination of DLI and blinatumomab might show some synergy (53). In a clinical trial, blinatumomab maintenance for high-risk or MRD positive patients was evaluated with significant benefit only in patients with an intact immune milieu. The median time from HCT to treatment was 78 days, probably correlating with tapering of immunosuppression (54). In contrast, Vγ9Vδ2 immunotherapy might be initiated earlier given the absence of alloreactivity and early reconstitution (14, 25). Higher γδ T cell counts correlate with a lower incidence of EBV reactivation (14). Preclinical data also suggests that γδ T cell precedes αβ T cell reconstitution by months (55), suggesting earlier utilization of this subset, e.g., in patients with MRD positivity after allogeneic stem cell transplantation.

Γδ T cell-based immunotherapy has certain disadvantages. Firstly, the fundamental principles of γδ T cell activation and exhaustion are not yet fully understood, which leads to some uncertainty regarding safety. Furthermore, the low abundance of γδ T cells might lead to unfavorable effector-target ratios that may limit therapeutic efficacy in patients with higher tumor burden. However, in patients with limited tumor burden, normal proportions of γδ T cells might be still sufficient to achieve acceptable effector: target ratios. Since γδ T cell expansion is suitable with a set of well-established and well-tolerated drugs such as zoledronic acid and interleukin-2, ex vivo expansion may be feasible (56). However, the efficacy of zoledronic acid on γδ T cell expansion *in vivo* has not been sufficiently studied (57). The bsAb presented here might also be used in addition to ex vivo-expanded γδ T cells or donor lymphocyte infusions to refine redirection against B-ALL blasts.

Further research is needed to address the risks and benefits of γδ T cell-based immunotherapy. Firstly, the efficacy of γδ T cell-targeting bsAbs against B-ALL should be proven in mice experiments, e.g., by utilization of surrogate molecules or transfer of human γδ T cells. Furthermore, safety of γδ T cell expansion and stimulation should be confirmed in animal experiments before concerting first-in-human trials.

In summary, we describe the first bispecific antibody for selective recruitment and activation of γδ T cells against CD19^+^ B-ALL blasts. Further evaluation of this bsAb *in vivo* should be performed to ultimately evaluate sustained tumor control, γδ T cell persistence, functional exhaustion and survival outcomes over time upon γδ T cell-based immunotherapy of B-ALL.

## Data Availability

The original contributions presented in the study are included in the article/**Supplementary Material**. Further inquiries can be directed to the corresponding author.
