# Supplementary material for: Generation and preclinical characterization of a novel bispecific CD19-TCRgammadelta antibody for the treatment of B cell acute lymphoblastic leukemia
Source: Front Immunol. 2026 Feb 27;17:1728424. doi: 10.3389/fimmu.2026.1728424 (PMC12982053; doi:10.3389/fimmu.2026.1728424)
Supplement: Supplementary file 1 [file Presentation1.pdf]

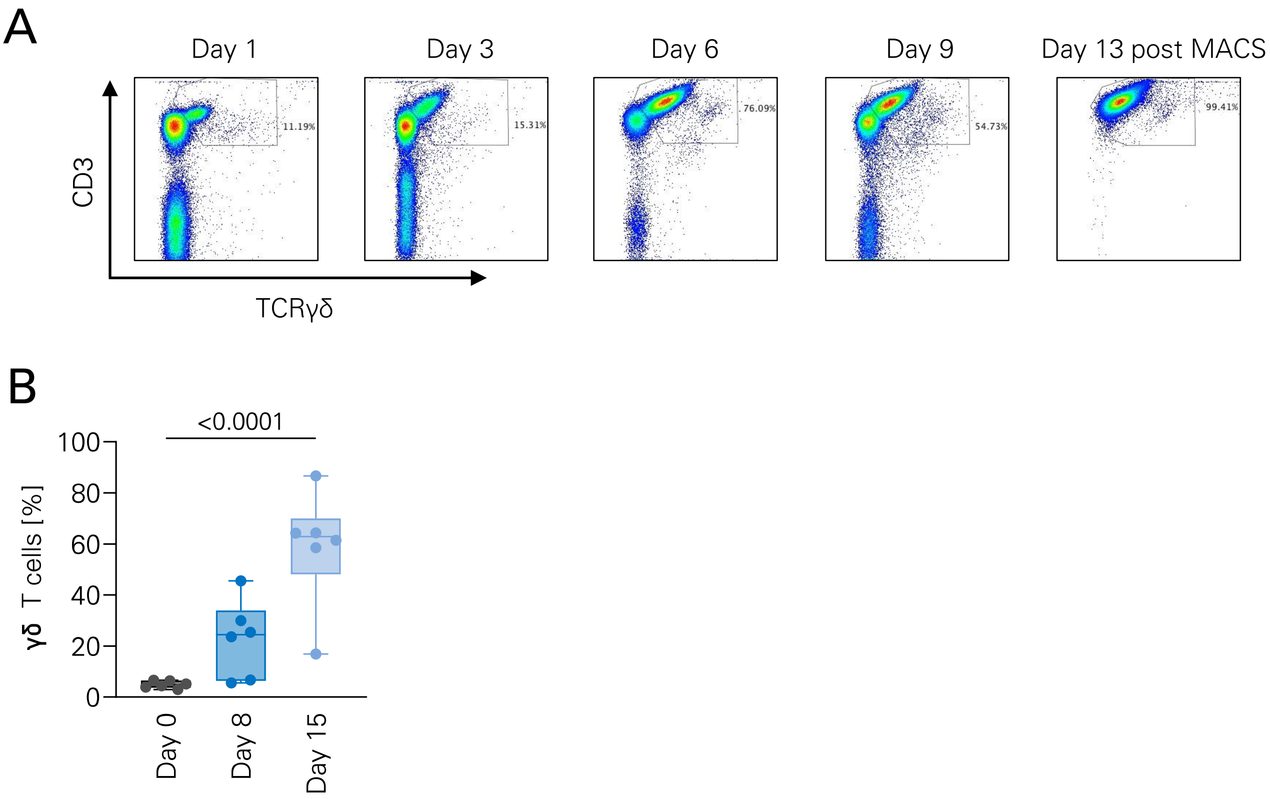

SUPPLEMENTARY FIGURE 1. Ex vivo expansion of human yd T cells

(A) yd T cell expansion using zolendronic acid and IL-2 for 13 days. Exemplary flow cytometry data from one out of 6 donors are depicted. On day 13, magnetic activated cell sorting (MACS) was performed.

(B) Percentage of yd T cells upon treatment with zolendronic acid and IL-2 (n=6).

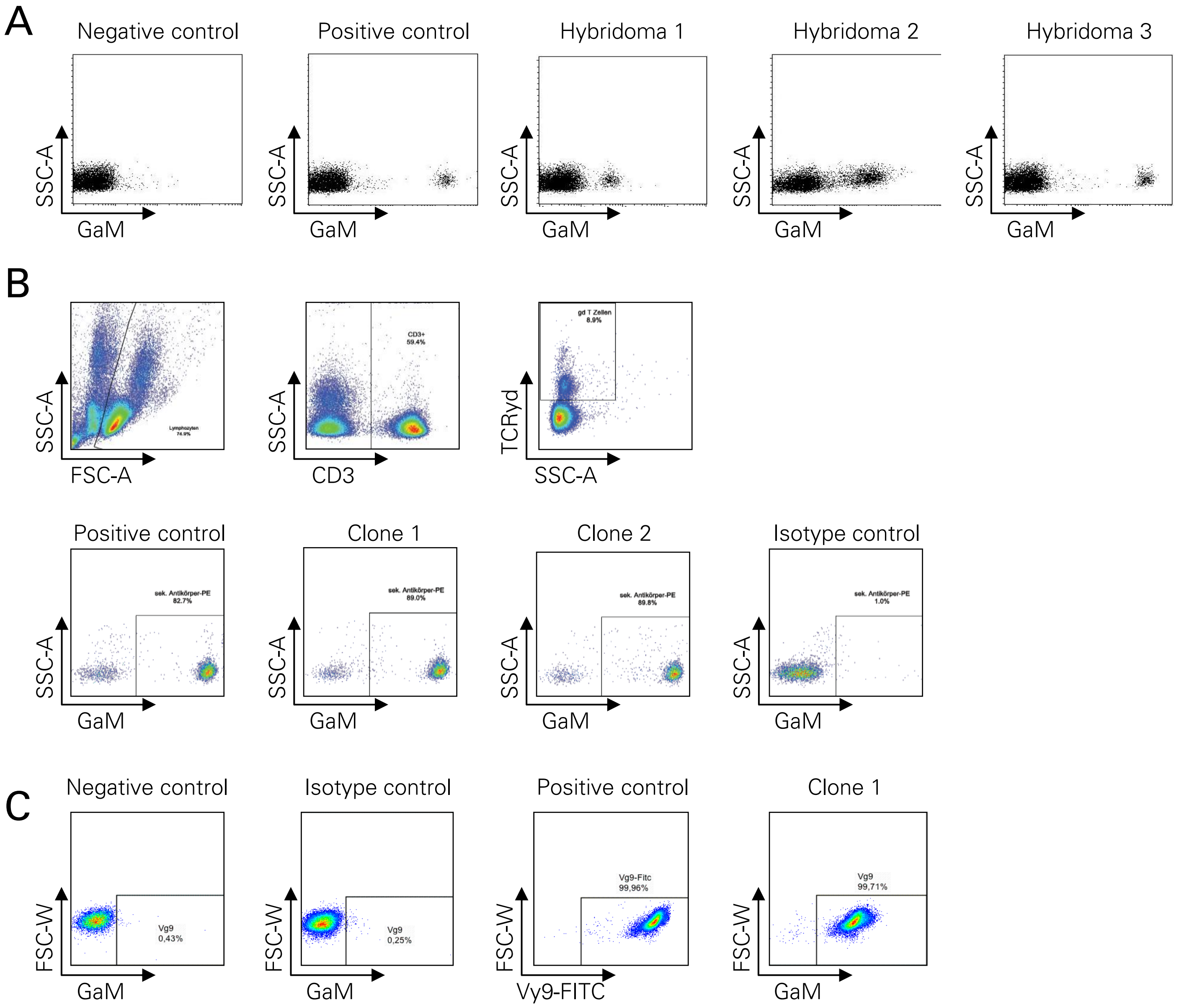

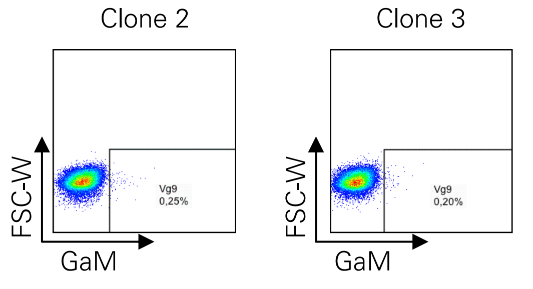

D

Clone	Heavy chain	Light chain	Specificity
**1**	**IgG1**	**kappa**	**Vγ9**
2	IgG1	n.d.	Vδ2
3	IgG1	n.d.	Vδ2
4	IgG1	n.d.	Vδ2
5	IgG1	n.d.	Vδ2
6	IgG1	n.d.	Vδ2
7	IgG1	n.d.	Vδ2

SUPPLEMENTARY FIGURE 2. Characterization of novel TCRγδ binding antibodies derived from hybridoma technology.

(A) Results from a flow cytometric binding analysis are depicted. A human anti-FAP antibody and an anti-human-Vδ2 antibody served as negative and positive control, respectively. Results derived from n=3 different hybridomas are shown.

(B) A flow cytometric binding analysis of the different monoclonal antibodies on human γδ T cells is depicted. Γδ T cells were gated as follows: Cells (FSC/SSC), T cells (CD3+), γδ T cells. Respresentative results from two different clones are depicted.

(C) Binding of the respective antibodies to a Vγ9Vδ1 T cell clone was measured by flow cytometry. Clone 1 exhibits binding to Vγ9Vδ1 T cells, confirming Vγ9 specificity.

(D) An overview of the seven generated murine monoclonal antibodies against Vγ9Vδ2 is shown. N.d., not determined.

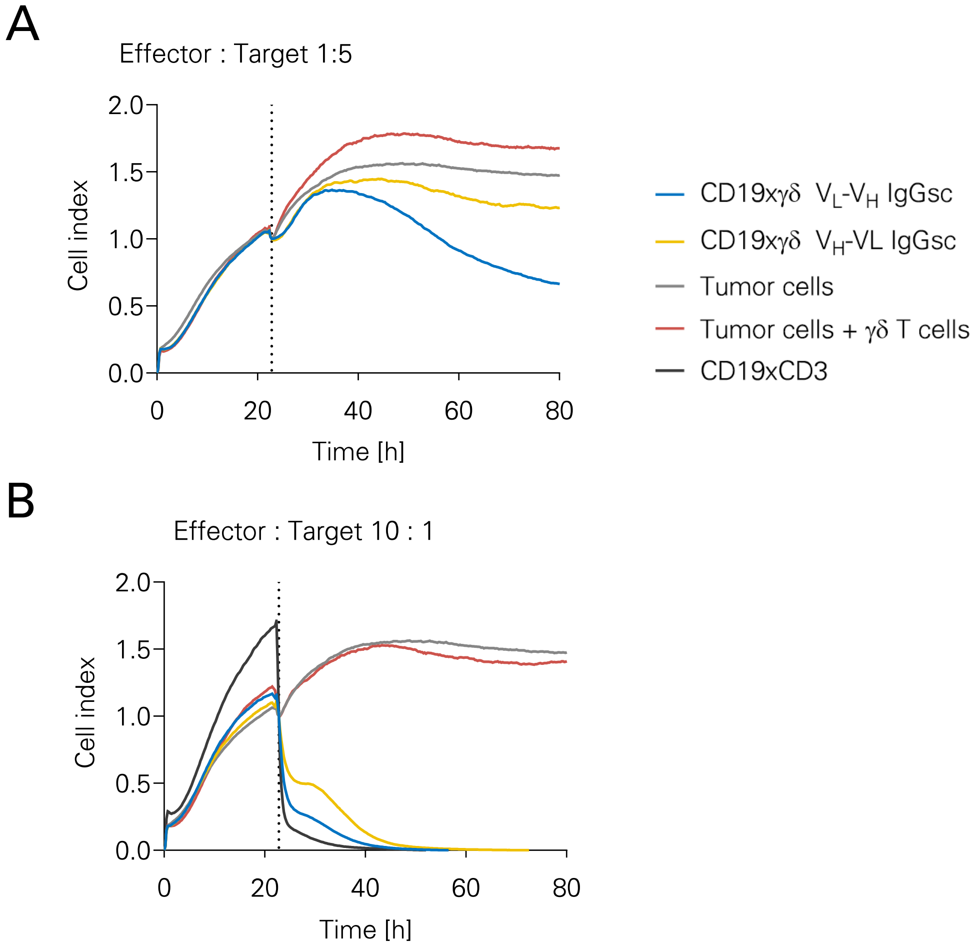

SUPPLEMENTARY FIGURE 3. Real-time lysis of CD19-transfected adherent tumor cells by CD19x γδ bispecific antibodies.

(A) xCELLigence real-time lysis assays were performed using CD19-transfected adherent MCF-7 cells. Twenty-four hours after tumor cell seeding, isolated γδ T cells (at effector:target ratios of (**A**) 1:5 and (**B**) 10:1) and bsAb at 10nm were added. Cell indices, which correspond to absolute numbers of viable tumor cells, were determined every 15 minutes

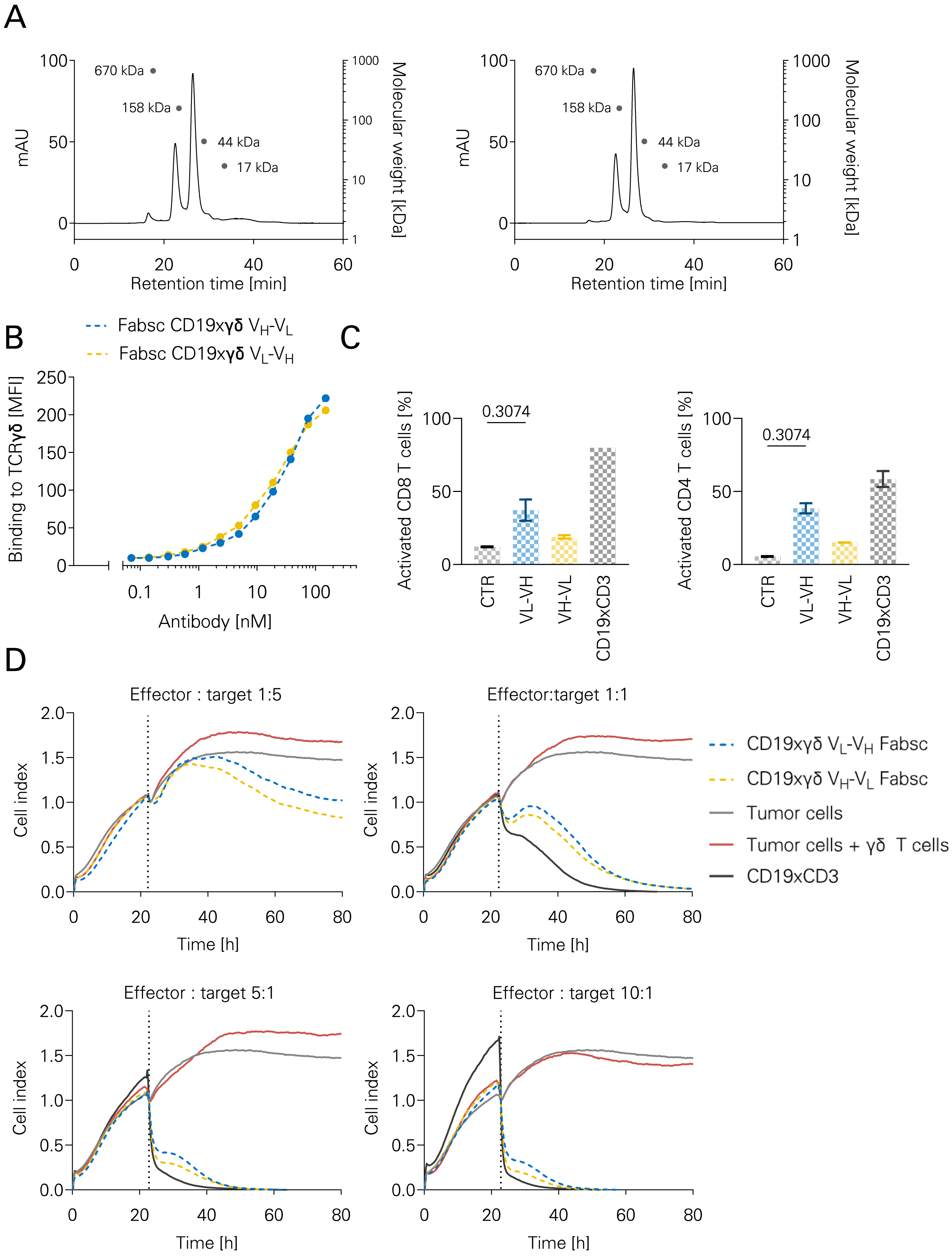

A
670 kDa
158 kDa
44 kDa
17 kDa
mAU
Molecular weight [kDa]
Retention time [min]
B
Fabsc CD19xγδ VH-VL
Fabsc CD19xγδ VL-VH
Binding to TCRγδ [MFI]
Antibody [nM]
C
Activated CD8 T cells [%]
Activated CD4 T cells [%]
0.3074
CTR
VL-VH
VH-VL
CD19xCD3
D
Effector : target 1:5
Effector:target 1:1
Effector : target 5:1
Effector : target 10:1
Cell index
Time [h]
CD19xγδ VL-VH Fabsc
CD19xγδ VH-VL Fabsc
Tumor cells
Tumor cells + γδ T cells
CD19xCD3

E

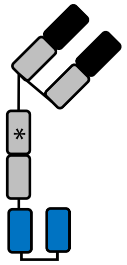

SUPPLEMENTARY FIGURE 4. Production and characterization of novel bispecific CD19xTCRyd antibodies in the Fabsc format.

(A) Size exclusion chromatography of the CD19xγδ VH-VL Fabsc bsAb (left) and the CD19xγδ VL-VH Fabsc bsAb (right).

(B) Human isolated γδ T cells were incubated with increasing concentrations of the indicated bsAb, followed by a goat-anti-human PE conjugate. Binding of the constructs to the human TCRγδ was analyzed by flow cytometry. MFI, mean fluorescence intensity.

(C) Human PBMCs and human isolated γδ T cells (ratio 5:1) of the same donors (n=3) were incubated for 48 hours in the presence of the respective bsAb (CD19xγδ VH-VL, CD19xγδ VL-VH, CD19xCD3, PSMAxγδ VH-VL as isotype control). CD4 and CD8 T cell activation was measured with flow cytometry. n=3, single values, bar diagram, Kruskal-Wallis test, Dunn's multiple comparisons.

(D) xCELLigence real-time lysis assays were performed using CD19-transfected adherent MCF-7 cells. Twenty-four hours after tumor cell seeding, isolated γδ T cells (at the indicated effector:target ratios) and bsAb at 10nm were added. Cell indices, which correspond to absolute numbers of viable tumor cells, were determined every 15 minutes.

(E) Schematic representation of the anti-CD19/CD20/PSMAxTCRVγ9 bispecific antibodies (bsAb) in the Fabsc format. Asterisks indicate a silenced CH2 domain to prevent FcγR binding.

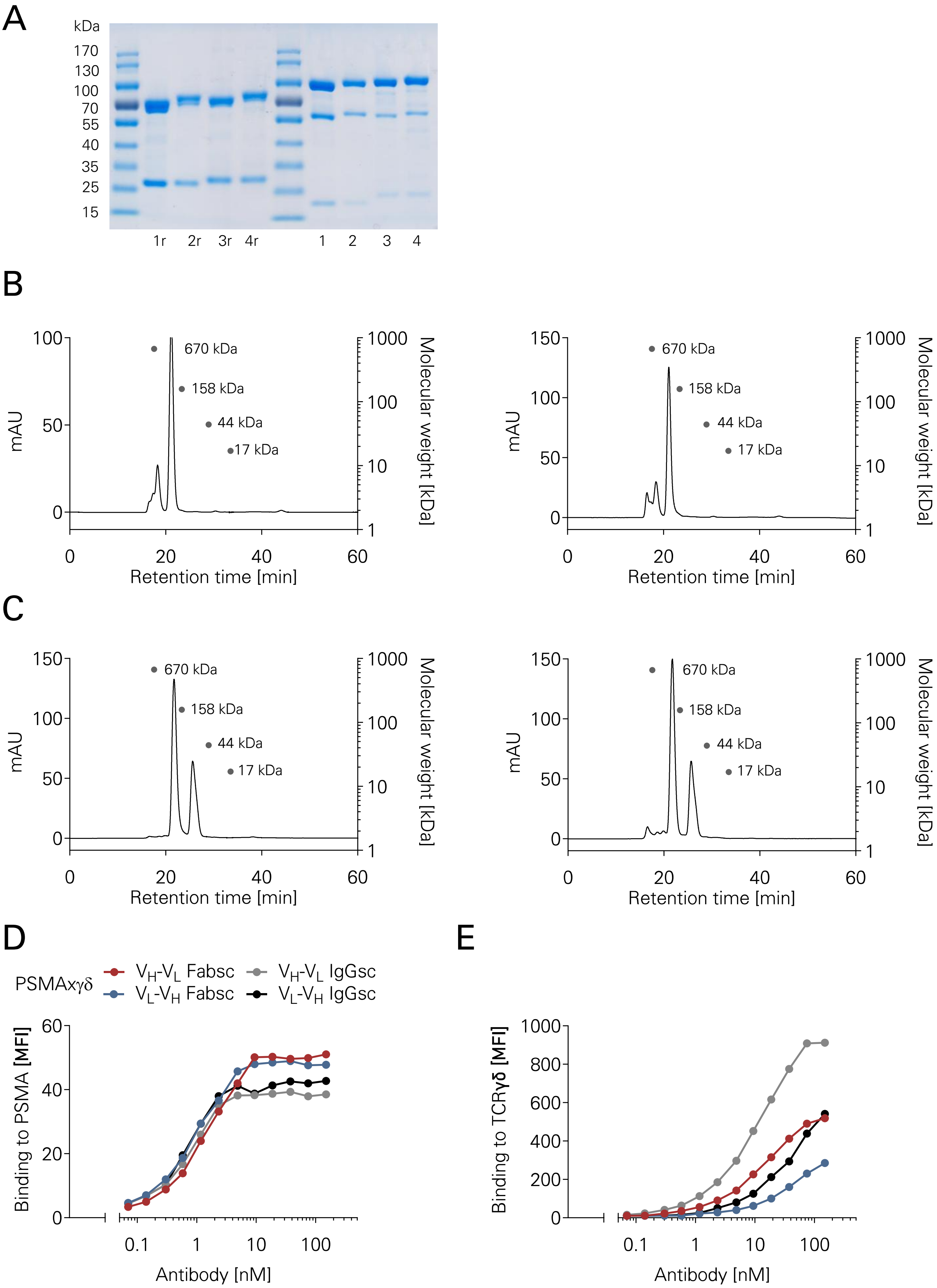

A
kDa
170
130
100
70
55
40
35
25
15
1r 2r 3r 4r 1 2 3 4
B
100
50
0
mAU
670 kDa
158 kDa
44 kDa
17 kDa
1000
100
10
1
Molecular weight [kDa]
0 20 40 60
Retention time [min]
150
100
50
0
mAU
670 kDa
158 kDa
44 kDa
17 kDa
1000
100
10
1
Molecular weight [kDa]
0 20 40 60
Retention time [min]
C
150
100
50
0
mAU
670 kDa
158 kDa
44 kDa
17 kDa
1000
100
10
1
Molecular weight [kDa]
0 20 40 60
Retention time [min]
150
100
50
0
mAU
670 kDa
158 kDa
44 kDa
17 kDa
1000
100
10
1
Molecular weight [kDa]
0 20 40 60
Retention time [min]
D
PSMAxγδ
V_H-V_L Fabsc
V_L-V_H Fabsc
V_H-V_L IgGsc
V_L-V_H IgGsc
60
40
20
0
Binding to PSMA [MFI]
0.1 1 10 100
Antibody [nM]
E
1000
800
600
400
200
0
Binding to TCRγδ [MFI]
0.1 1 10 100
Antibody [nM]

SUPPLEMENTARY FIGURE 5. Generation and characterization PSMAxTCRyd antibodies.

(A) Schematic representation of the anti-CD19/CD20/PSMAxTCRVγ9 bispecific antibodies (bsAb) in the Fabsc format. Asterisks indicate a silenced CH2 domain to prevent FcγR binding.

(B) SDS analysis of four different bsAb in the Fabsc format (1, CD19xγδ VL-VH; 2, PSMAxγδ VL-VH; 3, CD19xγδ VH-VL; 4, PSMAxγδ VH-VL). Columns marked with ‚r‘ indicate reducing conditions.

(C) Size exclusion chromatography of the IgGsc PSMAxγδ VH-VL (left) and IgGsc PSMAxγδ VL-VH (right) bsAb.

(D) Size exclusion chromatography of the Fabsc PSMAxγδ VH-VL (left) and Fabsc PSMAxγδ VL-VH (right) bsAb.

(E) Human PSMA+ prostate carcinoma cells 22Rv1 were incubated with increasing concentrations of the indicated bsAb, followed by a goat anti-human PE conjugate. Binding of the bsAb to human PSMA was analyzed by flow cytometry. MFI, mean fluorescence intensity.

(F) Human isolated γδ T cells were incubated with increasing concentrations of the indicated bsAb, followed by a goat-anti-human PE conjugate. Binding of the constructs to the human TCRγδ was analyzed by flow cytometry. MFI, mean fluorescence intensity.

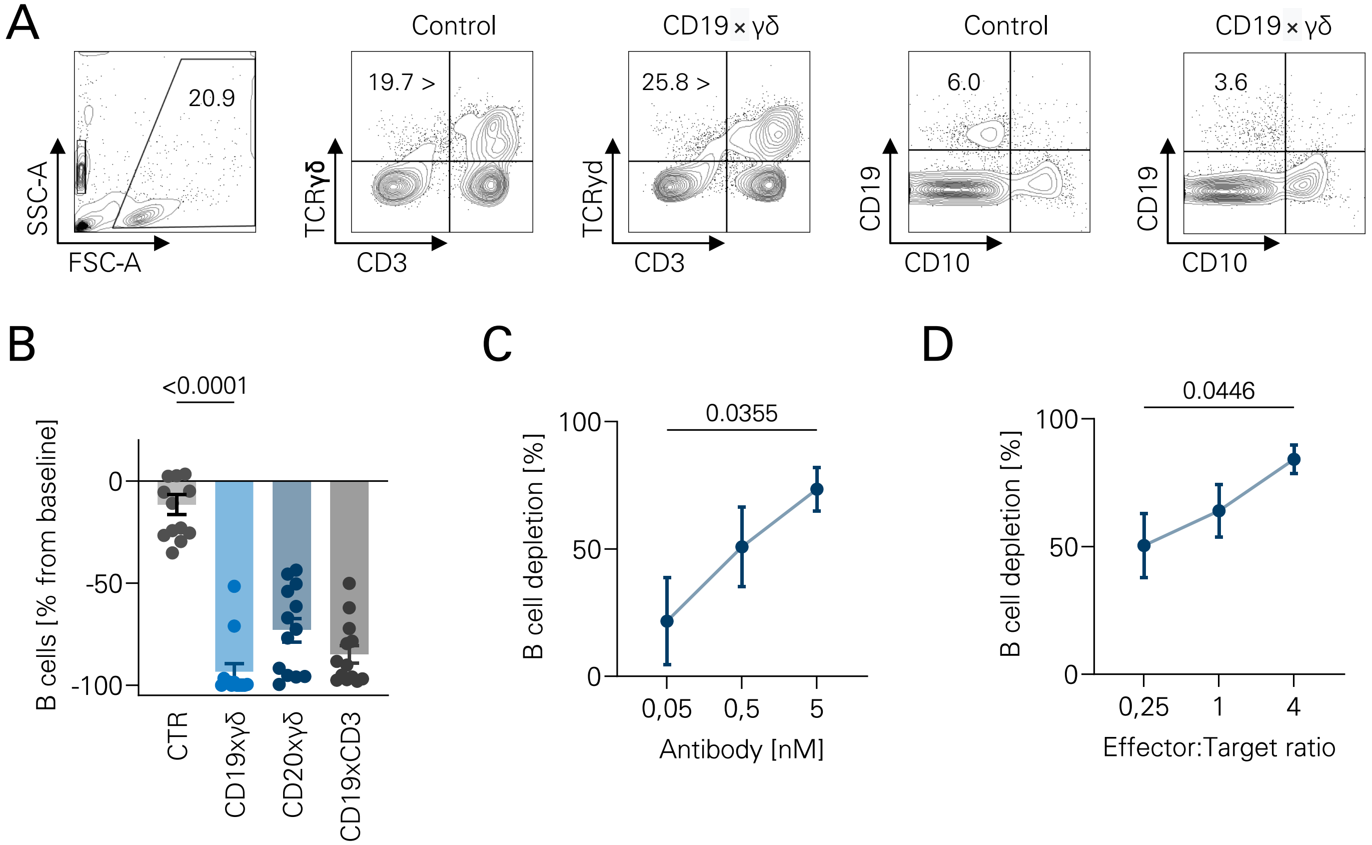

SUPPLEMENTARY FIGURE 6 Lysis of primary human B cells by preactivated yd T cells and CD19/CD20xyd bsab.

(A) An exemplary gating strategy for autologous B cell lysis assays is pictured: cells (FSC/SSC), singlets (FSC/FSC; not shown), B cells ($CD10^{-}CD19^{+}$), γδ T cells ($CD3^{+}TCR\gamma\delta^{+}$)

(B) Isolated γδ T cells and PBMC containing B cells were cultured in the presence of the indicated bsAb for 96 hours. Flow cytometric analysis was performed to determine absolute tumor cell numbers. A MOPCxγδ bsAb at E:T ratio of 1:1 served as negative control (CTR). n=8 single values, bar diagram, ANOVA with Bonferroni correction.

(C) B cell lysis assays were performed as described in A. The CD20xγδ antibody was added at the indicated concentrations. n=8, mean + SEM. ANOVA with Bonferroni correction

(D) B cell lysis assays were performed as described in A using the CD20xγδ antibody at 5nM. Isolated γδ T cells were added at the indicated effector:target ratios. n=11. mean + SEM, ANOVA with Bonferroni correction

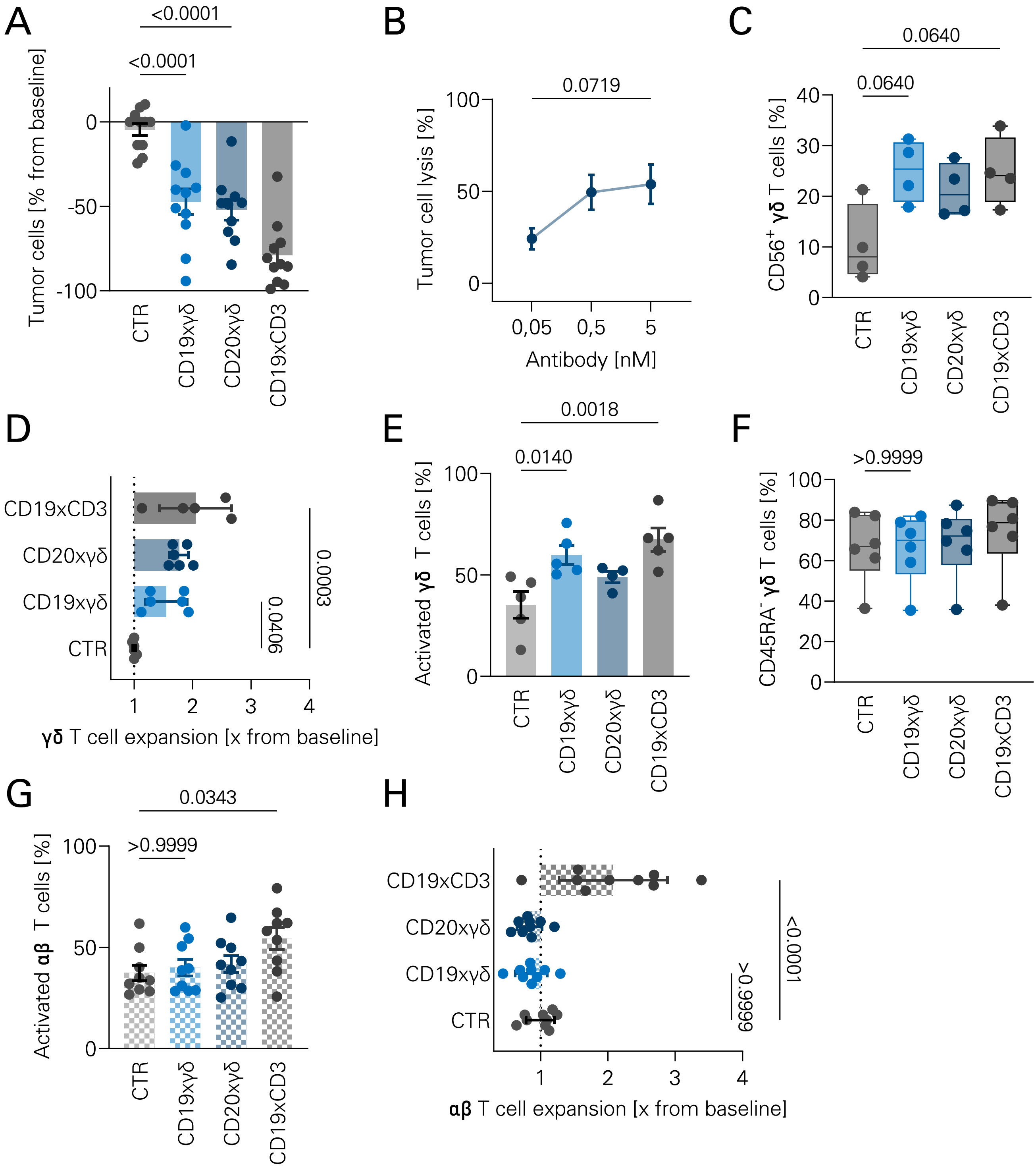
A
<0.0001
<0.0001
Tumor cells [% from baseline]
0
-50
-100
CTR
CD19xγδ
CD20xγδ
CD19xCD3
B
Tumor cell lysis [%]
100
50
0
0.0719
0,05
0,5
5
Antibody [nM]
C
0.0640
0.0640
CD56+ γδ T cells [%]
40
30
20
10
0
D
CD19xCD3
CD20xγδ
CD19xγδ
CTR
0.0003
0.0406
1
2
3
4
γδ T cell expansion [x from baseline]
E
0.0018
0.0140
Activated γδ T cells [%]
F
>0.9999
CD45RA- γδ T cells [%]
80
60
40
20
G
0.0343
>0.9999
Activated αβ T cells [%]
H
<0.0001
>0.9999
αβ T cell expansion [x from baseline]

SUPPLEMENTARY FIGURE 7 Subset-restricted activation of yd T cells against the leukemic cell line Nalm-16.

(A) Isolated γδ T cells and Nalm-16 at an effector:target ratio of 1:1 were cultured in the presence of the indicated bsAb for 72 hours. Flow cytometric analysis was performed to determine absolute tumor cell numbers. A MOPCxγδ bsAb served as negative control (CTR). n=11 single values, bar diagram, ANOVA with Bonferroni correction.

(B) Flow cytometric lysis assays were performed as described in (A) using an effector:target ratio of 1:1. The CD20xγδ bsAb was added at the indicated concentrations. n=5, mean + SEM, Kruskal-Wallis test, Dunn's multiple comparisons.

(C) Isolated γδ T cells and Nalm-16 at an effector:target ratio of 1:1 were cultured in the presence of the indicated bsAb for 72 hours. CD56 expression on γδ T cells was measured by flow cytometry. n=5, boxplots with min/max whiskers. Kruskal-Wallis test, Dunn's multiple comparisons.

(D) Isolated γδ T cells and Nalm-16 cells were cultured with different bsAb at 5nM for 72 hours. Γδ T cell expansion was determined using absolute cell numbers after the indicated treatments divided by absolute cell numbers of untreated PBMC. n=5-6, single values, bar diagram, ANOVA with Bonferroni correction.

(E) CD69 expression as surrogate marker for activation status was measured on γδ T cells after incubation with different bsAb and Nalm-16 cells for 72 hours. n=5, single values, bar diagram, ANOVA with Bonferroni correction.

(F) CD45Ra negativity as surrogate marker for memory cells was measured on γδ T cells after incubation with different bsAb and Nalm-16 for 72 hours. n=6, boxplots with min/max whiskers, bar diagram, ANOVA with Bonferroni correction.

(G) CD69 expression as surrogate marker for activation status was measured on αβ T cells after incubation with different bsAb for 72 hours. n=9, single values, bar diagram, ANOVA with Bonferroni correction.

(H) Human PBMC were cultured with different bsAb at 5nM for 72 hours. αβ T cell expansion was determined using absolute cell numbers after the indicated treatments divided by absolute cell numbers of untreated PBMC. n=9, single values, bar diagram, ANOVA with Bonferroni correction.

A

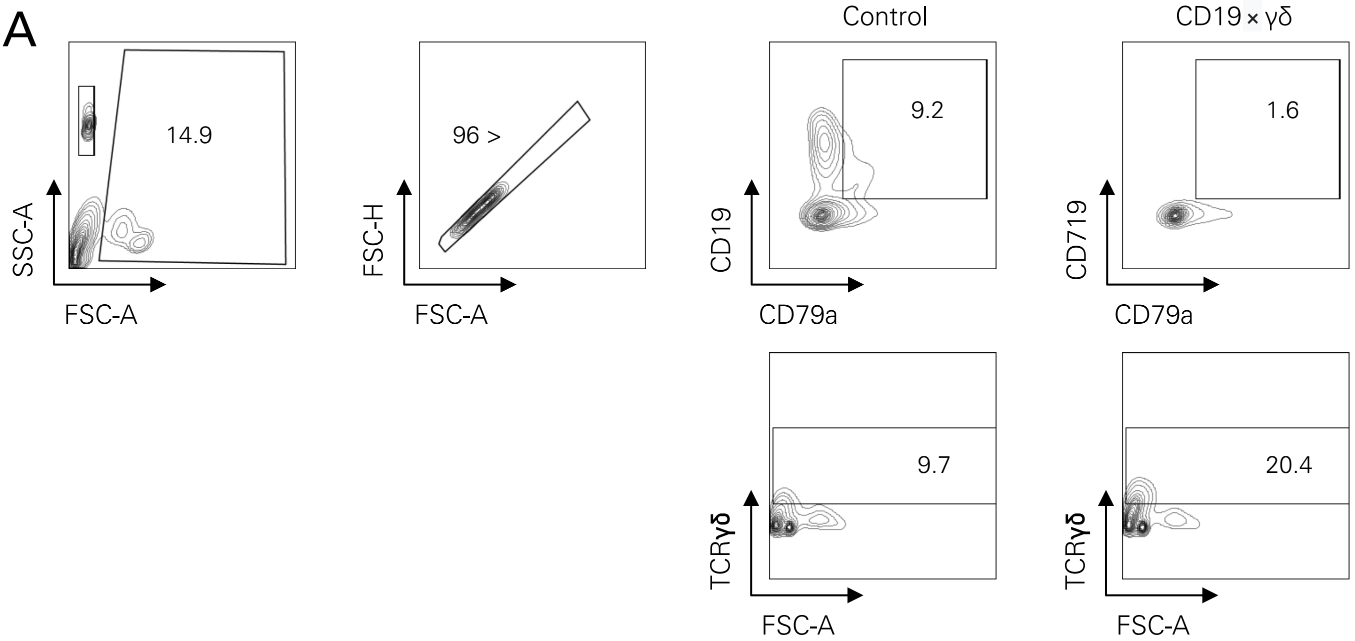

B

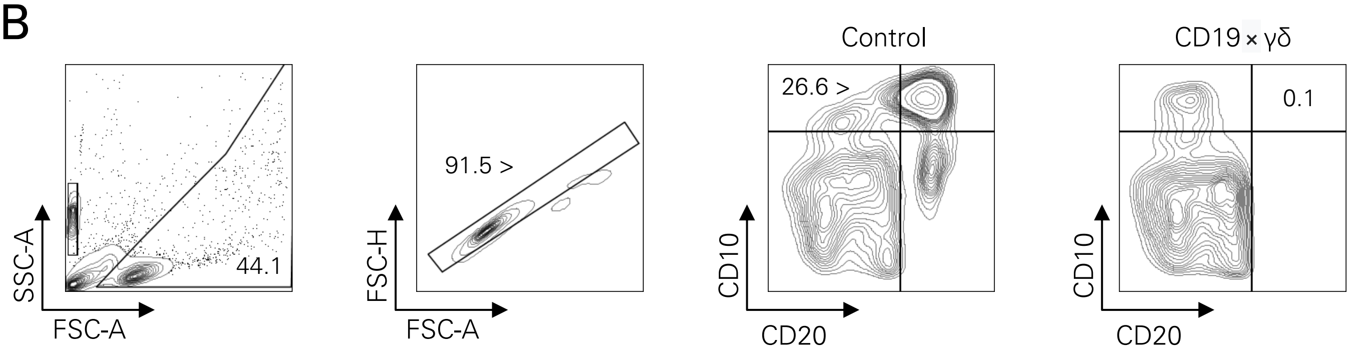

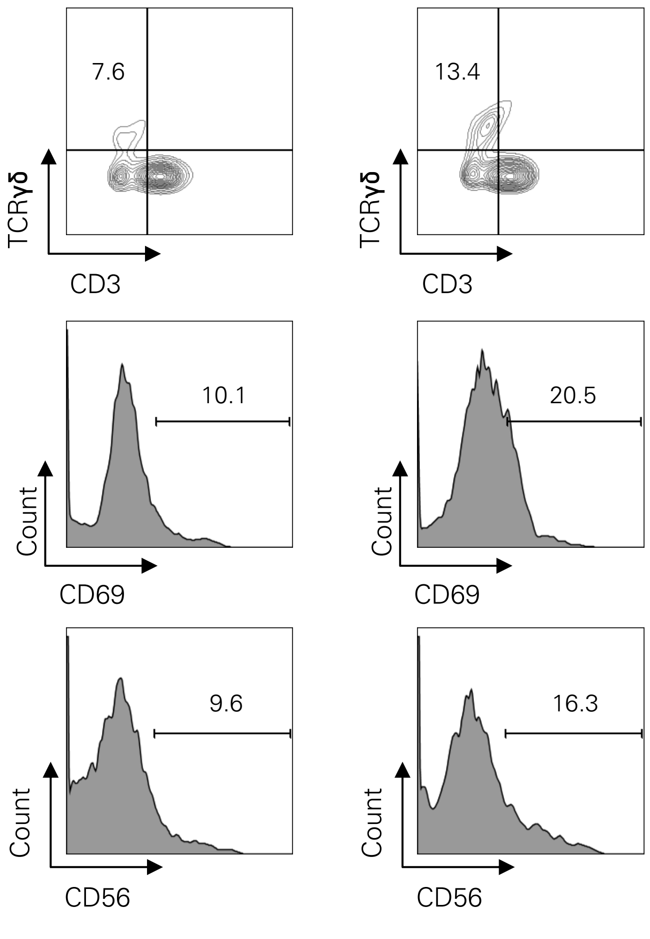

SUPPLEMENTARY FIGURE 8 Gating strategies in flow cytometry based lysis assays.

(A) An exemplary gating strategy for flow cytometry based lysis assays with isolated γδ T cells and human primary leukemic blasts is pictured: cells (FSC/SSC), singlets (FSC/FSC), B cells ($CD10^{-}CD19^{+}$), blasts (e.g.,$CD19^{+}CD79a^{+}$), γδ T cells ($TCR\gamma\delta^{+}$).

(B) An exemplary gating strategy for flow cytometric lysis assays with with isolated γδ T cells and the human ALL cell line Nalm-16 is pictured: cells (FSC/SSC), singlets (FSC/FSC), B cells ($CD10^{-}CD19^{+}$) or Nalm-16 ($CD10^{+}CD19^{+}$), γδ T cells ($CD3^{+}TCR\gamma\delta^{+}$), $CD69^{+}$ (histograms), $CD56^{+}$ (histograms).

A

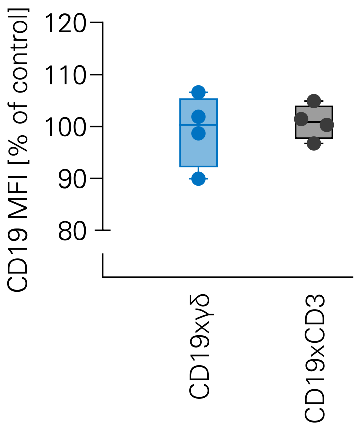

SUPPLEMENTARY FIGURE 9 CD19 antigen escape upon bsAb treatment

(A) CD19 mean fluorescence intensity (MFI) of $CD10^{-}CD19^{+}$, primary leukemic blasts after 96h treatment with blinatumomab or CD19xγδ (% of control, untreated blasts)

Target	Conjugate	Manufacturer	Catalogue number
TCR γδ	FITC	BioLegend	306708
TCRab	PE	BioLegend	331208
CD3	PerCP/Cy5.5	BioLegend	317336
CD4	AF700	BioLegend	300526
CD8a	APC/Cy7	BioLegend	300912
CD10	APC/Cy7	BioLegend	312212
CD19	PE	BioLegend	302208
CD20	APC	BioLegend	302310
CD34	APC	BioLegend	343607
CD45RA	PerCP	BioLegend	304155
CD56	PE/Cy5.5	BioLegend	362515
CD69	PE/Cy7	BioLegend	310912
CD79a	APC	BioLegend	333505

SUPPLEMENTARY TABLE 1 Conjugated antibodies used in flow cytometry staining.
